# Deeper Insights on *Alchornea cordifolia* (Schumach. & Thonn.) Müll.Arg Extracts: Chemical Profiles, Biological Abilities, Network Analysis and Molecular Docking

**DOI:** 10.3390/biom11020219

**Published:** 2021-02-04

**Authors:** Kouadio Ibrahime Sinan, Gunes Ak, Ouattara Katinan Etienne, József Jekő, Zoltán Cziáky, Katalin Gupcsó, Maria João Rodrigues, Luisa Custodio, Mohamad Fawzi Mahomoodally, Jugreet B. Sharmeen, Luigi Brunetti, Sheila Leone, Lucia Recinella, Annalisa Chiavaroli, Giustino Orlando, Luigi Menghini, Massimo Tacchini, Claudio Ferrante, Gokhan Zengin

**Affiliations:** 1 Physiology and Biochemistry Research Laboratory, Department of Biology, Science Faculty, Selcuk University, Konya 42130, Turkey; sinankouadio@gmail.com (K.I.S.); akguneselcuk@gmail.com (G.A.); gokhanzengin@selcuk.edu.tr (G.Z.); 2 Laboratoire de Botanique, UFR Biosciences, Université Félix Houphouët-Boigny, 01 Abidjan, Ivory Coast; katinan.etienne@gmail.com; 3 Agricultural and Molecular Research and Service Institute, University of Nyíregyháza, 4400 Nyíregyháza, Hungary; jjozsi@gmail.com (J.J.); cziaky.zoltan@nye.hu (Z.C.); 4 Sotiva Seed Ltd., 4440 Tiszavasvári, Hungary; sotiva@sotiva.hu; 5 Centre of Marine Sciences, Faculty of Sciences and Technology, University of Algarve, Ed. 7, Campus of Gambelas, 8005-139 Faro, Portugal; mary_p@sapo.pt (M.J.R.); lcustodio@ualg.pt (L.C.); 6 Department of Health Sciences, Faculty of Medicine and Health Sciences, University of Mauritius, Réduit 80837, Mauritius; f.mahomoodally@uom.ac.mu (M.F.M.); sharmeenjugs@gmail.com (J.B.S.); 7 Department of Pharmacy, G. d’Annunzio University, via dei Vestini n. 31, 66100 Chieti, Italy; luigi.brunetti@unich.it (L.B.); sheila.leone@unich.it (S.L.); lucia.recinella@unich.it (L.R.); annalisa.chiavaroli@unich.it (A.C.); giustino.orlando@unich.it (G.O.); luigi.menghini@unich.it (L.M.); 8 Department of Life Sciences and Biotechnology (SVeB), UR7 Terra&Acqua Tech, University of Ferrara, 44121 Ferrara, Italy

**Keywords:** *Alchornea cordifolia*, antioxidant, enzyme inhibition, chemical profile, cytotoxicity, bioinformatics

## Abstract

*Alchornea cordifolia* (Schumach. & Thonn.) Müll. Arg. is a well-known African medicinal plant traditionally used for various healing purposes. In the present study, methanolic, ethyl acetate and infusion extracts of *A. cordifolia* leaves were studied for their total phenolic and flavonoid contents and screened for their chemical composition. Moreover, the enzyme (acetyl- and butyryl-cholinesterases, α-amylase, α-glucosidase, and tyrosinase) inhibitory and cytotoxicity activities on HepG2: human hepatocellular carcinoma cells, B16 4A5: murine melanoma cells, and S17: murine bone marrow (normal) cells of extracts were evaluated. Finally, components-targets and docking analyzes were conducted with the aim to unravel the putative mechanisms underlying the observed bio-pharmacological effects. Interestingly, the infusion and methanolic extracts showed significantly higher total phenolic and flavonoid contents compared with the ethyl acetate extract (TPC: 120.38–213.12 mg GAE/g and TFC: 9.66–57.18 mg RE/g). Besides, the methanolic extracts followed by the infusion extracts were revealed to contain a higher number of compounds (84 and 74 compounds, respectively), while only 64 compounds were observed for the ethyl acetate extract. Gallic acid, ellagic acid, shikimic acid, rutin, quercetin, myricetin, vitexin, quercitrin, kaempferol, and naringenin were among the compounds that were commonly identified in all the studied extracts. Additionally, the methanolic and infusion extracts displayed higher antioxidant capacity than ethyl acetate extract in all assays performed. In ABTS and DPPH radical scavenging assays, the methanol extract (500.38 mg TE/g for DPPH and 900.64 mg TE/g for ABTS) exhibited the best ability, followed by the water and ethyl acetate extracts. Furthermore, the extracts exhibited differential enzyme inhibitory profiles. In particular, the methanolic and infusion extracts showed better cytotoxic selectivity activity against human hepatocellular carcinoma cells. Overall, this study demonstrated *A cordifolia* to be a species worthy of further investigations, given its richness in bioactive phytochemicals and wide potentialities for antioxidants and pharmacological agents.

## 1. Introduction

*Alchornea cordifolia* (Schumach. & Thonn.) Müll. Arg. belonging to the Euphorbiaceae family is found generally in African regions, and is traditionally used for the treatment of a number of fungal, bacterial, parasitic, and inflammatory disorders [1]. *A. cordifolia* leaves are used to treat wounds, sores, and cuts [2]. Moreover, the plant has been documented to be used for treating conditions like coughs, headaches, colds, for control of spontaneous abortion, as well as for the control and relief of asthmatic attacks [3]. Both root and stem barks are used in the treatment of jaundice and the powdered leaves of *A. cordifolia* are utilized to cure wounds and diarrhea. Additionally, the treatment of gastrointestinal and urinary disorders forms part of its traditional usage. The leaves and root bark are also employed to alleviate leprosy and as an antidote to snake venom. Besides, the fruit is applied to treat eye and pigmentation problems, while a decoction of leafy twigs is applied to remedy fever, rheumatic pains, and malaria [4].

Several reports on the biological activities of *A. cordifolia* have shown the plant to possess antiinflammatory, antidiarrhoeal, hepaprotective, antiviral, and antidiabetic properties [5,6,7,8,9]. In addition, various antimicrobial screenings of *A. cordifolia* have revealed its effectiveness against a wide range of pathogenic microbes including gastrointestinal, skin, respiratory, and urinary tract pathogens, thus supporting the traditional use of the plant for the treatment of such ailments [10,11,12,13]. These significant pharmacological actions have been linked to several active principles isolated from the different parts of *A. cordifolia*. For instance, polysaccharide fractions isolated from *A. cordifolia* demonstrated a potent immunomodulatory effect through the activation of human and murine monocyte/macrophages, resulting in modulation of nitric oxide and cytokine production, thereby enhancing resistance to infection. Furthermore, previous phytochemical investigations of *A. cordifolia* showed the plant to be mostly rich in alkaloids, fatty acids, terpenoids, steroids, flavonoids, and phenolic acids [14]. The main groups of phytochemicals in *A. cordifolia* parts contained several compounds. For example, several studies reported the presence of nonacosane, oleic acid [15], octadecanal [16], and octen-3-ol [17] as fatty acids. Regarding phenolic compounds, gallic acid, protocatechuic acid [18,19], hypericin, and quercetin [19] were found in the extracts from *A. cordifolia* parts. Yohimbine [20], Alchorneine [18], and triisopentenyl guanidine [21] were reported as alkaloids. Volatile components such as methyl salicylate (25.3%), citronellol (21.4%), α-phellandrene (7.4%), terpinolene (5.7%), and 1,8-cineole (5.5%) have also been identified in the essential oil of *A. cordifolia* fruits, exhibiting antimicrobial activities [17].

It is broadly acknowledged that natural products have contributed massively to contemporary drug development. Although the popularity of the synthetic products increased owing to their time effectiveness, production cost, easy quality control, rigorous regulation, and rapid effects, their safety and efficacy have nevertheless always remained questionable, resulting in the dependence of more than 80% of the total population in the developing world on natural products, due to their time tested safety and effectiveness. A huge number of natural product-derived compounds at different stages of clinical development suggest the existing feasibility and importance of the use of natural products as sources of novel drug candidates [22].

Hence, given the broad spectrum of traditional applications and multiple pharmacological properties attributed to *A. cordifolia* as a medicinally important plant, the present research was guided to further investigate the biological potential of this species. Therefore, along with the screening of the bioactive constituents, the antioxidant, enzyme inhibitory, and cytotoxic properties were assessed on extracts prepared using different solvents (ethyl acetate, methanol, and water) and extraction techniques (maceration and infusion). Finally, components-targets and docking analyzes were conducted with the aim to predict the possible mechanisms between the observed bio-pharmacological effects.

## 2. Materials and Methods

### 2.1. Plant Material and Preparation of Extracts

*Alchornea cordifolia* samples were obtained in a field study at the village of Assanou (Yamoussoukro-Côte d’Ivoire). The plants materials were identified by one of the co-authors (Dr. Quattara Katinan, Etienne). The leaves from these plants were carefully separated and they were dried in a dark environment and ground by using a laboratory mill.

The plant extracts were obtained by using maceration and infusion techniques. Two organic solvents, namely methanol (MeOH), and ethyl acetate (EA) (5 gr plants) were stirred with 100 mL of one of the solvents at room temperature for 24 h in a magnetic stirrer. The ethyl acetate and methanol were renewed in the 12th hour in the extraction period. Infusion was obtained as follows: 5 gr of plant materials were suspended with 100 mL of boiled water. All extracts were filtered and then dried. They were stored at 4 °C until experimentation.

### 2.2. Profile of Bioactive Compounds

Spectrophotometric methods were used to determine total phenolic and flavonoid content as conducted in earlier papers. Standard equivalents (gallic acid equivalent: GAE for phenolic and rutin equivalent: RE for flavonoid) were used to explain the contents in the plant extracts [23].

The UHPLC/MS/MS technique was used to analyze the different extracts. Chromatographic separation was accomplished with a Dionex Ultimate 3000RS UHPLC instrument, equipped with a Thermo Accucore C18 (100 mm × 2.1 mm i. d., 2.6 μm) analytical column for the separation of compounds. Water (A) and methanol (B) containing 0.1% formic acid were both employed for mobile phases. The total run time was 70 min. A Thermo Q Exactive mass spectrometer was used to detect the separated components. All extracts were performed in two chromatographic runs with the recording of mass spectra in positive and negative ion mode, and protonated [M+H]^+^ or deprotonated molecules [M-H]^-^ and their fragments were recorded.The elution profile and all exact analytical conditions were published [24].

### 2.3. Determination of Antioxidant and Enzyme Inhibitory Effects

Different protocols were performed to explain antioxidant properties of *A. cordifolia* extracts. The protocols included reducing power (CUPRAC and FRAP), metal chelating, phosphomolybenum and free radical scavenging (DPPH and ABTS). Experimental details were given in our previous paper [23]. Inhibitory effects of *A. cordifolia* extracts were tested against different enzymes (tyrosinase, amylase, glucosidase and cholinesterase). Both antioxidant and enzyme inhibition assays were explained by using standard equivalents (trolox and EDTA for antioxidants; galatamine for cholinesterase; kojic acid for tyrosinase; acarbose for amylase and glucosidase).

### 2.4. Cell Culture

The HepG2 (human hepatocarcinoma) and S17 (murine bone marrow stromal) cell lines were offered by the Centre for Molecular and Structural Biomedicine, University of Algarve, Portugal), and B16 4A5 (mouse melanoma) cells were obtained from Sigma-Aldrich (Germany). All cell lines were cultivated in Dulbecco’s Modified Eagle medium (DMEM) supplemented with fetal bovine serum (10%), L-glutamine (2 mM), and penicillin (50 U/mL)/streptomycin (50 μg/mL), under a moistened environment at 37 °C and 5% of CO_2_.

### 2.5. Assessment of Cell Viability and Selectivity

Cells were seeded in 96-well microtitration plates at 5 × 10^3^ (HepG2 and S17) and 2 × 10^3^ (B16 4A5) cells per well, and incubated for 24 h. Afterwards, extracts were applied at a concentration of 100 μg/mL during 72 h. DMSO at 0.5% was used as a negative control. The percentage of viable cells was assessed by the MTT 3-(4,5-dimethylthiazol-2-yl)-2,5-diphenyltetrazolium bromide) test, and was calculated in relation to the negative control, as previously described [25]. Selectivity was determined by dividing the percentage of cellular viability of the non tumoral cell line (S17) by the corresponding value of the tumoral cell lines (HepG2 and B16 4A5).

### 2.6. Bioinformatics

Bioinformatics analysis was carried out according to recent studies of ours [26,27]. Briefly, the simplified molecular-input line-entry system (SMILES) of identified phytocompounds were run on bioinformatics platforms STITCH (http://stitch.embl.de/cgi/network.pl) and SwissTargetPrediction (http://www.swisstargetprediction.ch/), in order to build components-targets analysis. Non-bonding interactions between phytocompounds and target proteins identified by HPLC-MS and bioinformatics, respectively, were calculated by the virtual screening software Autodock Vina of PyRx 0.8 software. The protein data bank (PDB) structures of the target proteins were downloaded from the Protein Data Bank platform (www.wwpdb.org/) as follows: 1HCQ for estrogen receptor 1 (ESR1), 1QCF for hemopoietic cell kinase (HCK); 3BGZ for pim-1 oncogene (PIM-1); 5I3B for tyrosinase; 1GQR for acethylcholinesterase (AchE). Details about the docking calculations are fully described in our recent paper [28].

### 2.7. Statistical Analysis

All analysis was performed in triplicate and the results are depicted as mean ± SD. To detect differences among extracts, One-Way ANOVA (with Tukey’s test) was performed at *p* < 0.05. The statistical approach was done by using R software (version 3.6.2).

## 3. Results and Discussion

In recent times, much consideration has been given to characterization of phytochemical contents and evaluating the antioxidant activities of different plant-based extracts. In this regard, a number of extraction solvents has been reported to be used for extracting bioactive components from plant materials. Among them, water, ethanol, methanol, ethyl acetate, and acetone are the most commonly used ones [29]. Hence in the present work, the total phenolic and flavonoid contents of the extracts were measured using standard spectrophotometric assays, while the qualitative screening of the phytochemical content was done using mass spectrometric techniques.

For instance, based on the spectrophotometric assays, the extracts were found to be richer in phenolics than in flavonoids. The infusion extract, followed by the methanolic extracts yielded the highest total phenolic content (TPC), while the ethyl acetate extract contained a lesser amount of TPC. On the other hand, the methanolic followed by the infusion extracts were both seen to yield a relatively higher total flavonoid content (TFC) than ethyl acetate extract. Hence, water and methanol were clearly found to be better solvents in extracting bioactive compounds relative to ethyl acetate (Table 1).

Moreover, results from the mass spectrometric methods revealed that methanolic extract contains the highest number of compounds, notably 84 compounds, followed by the aqueous extract which contained 74 compounds. On the other hand, only 64 compounds were identified in the ethyl acetate extract (Table 2, Supplementary Tables S1–S3 and Supplementary Figures S1–S6). The extraction procedures and solvents are main factors for obtaining phytochemicals from plant materials [30] and they have different polarity properties. In particular, phenolic compounds with hydroxyl groups exhibited polar characteristics and therefore explained why methanol and water were better extracting solvents than ethyl acetate in the present study, in agreement in other studies [31].

Interestingly, gallic acid, ellagic acid, shikimic acid, vitexin, rutin, quercetin, myricetin, quercitrin, kaempferol, and naringenin were among the compounds commonly identified in all the studied extracts (Table 2). Many of these polyphenolic compounds have been extensively documented to possess important biological activities such as antioxidant, anti-inflammatory, and anti-tumoral activities, as well as to exert beneficial effects on cardiovascular and brain functions, amongst other effects [32,33,34,35,36,37,38,39].

Accumulating scientific evidence suggests that over-production of reactive oxygen species is linked to the risks of global health problems, including cardiovascular disease and cancer. Antioxidant compounds can prevent the deleterious effects of reactive oxygen species from developing and can be beneficial for health promotion, thus causing the research conducted in this area to develop rapidly [40]. Indeed, there are diverse in vitro methods to quantify the antioxidant activity of plant extracts [41]. In this study, a total of six methods were used, namely DPPH and ABTS radical scavenging assays, FRAP, CUPRAC, metal chelating, as well as phosphomolydenum assays.

Remarkably, the methanolic and infusion extracts were found to possess significantly higher antioxidant capacity relative to the ethyl acetate extract in all of the antioxidant assays performed. For instance, while the methanolic extract showed the most significant activity in radical scavenging assays as well as a metal chelating ability assay, the infusion extract, on the other hand, demonstrated relatively higher total antioxidant capacity in phosphomolybdenum assay and the most potent reducing power in CUPRAC and FRAP assays. Nevertheless, notable radical scavenging potential was also observed by the infusion extract. Similarly, the methanolic extract was also found to be a strong reducing agent, as noted in the CUPRAC and FRAP assays (Table 3).

Various *A. cordifolia* extracts have also been evaluated for their antioxidative activities. For instance, Osei Akoto et al. [4] showed methanol, petroleum ether, and chloroform extracts to exhibit antioxidant potential tested using different assays. In their study, DPPH radical scavenging assay was performed and the IC_50_ values varied from 93.02 to 105.40 µg/mL. In addition, total antioxidant capacity was reported as 25.85–40.08 g ascorbic acid equivalent (AAE)/100 g.

Interestingly, the results obtained herein were in disagreement with that reported in the study of Kouakou–Siransy et al. [42], whereby the ethyl acetate extract was found to yield higher total phenols than the aqueous extract, as well as better scavenging activity. On the other hand, Barchan et al. [31] showed that water and methanol (polar solvents) extracts were almost equal to positive control BHT, whereas hexane and dichloromethane (non-polar solvents) extracts displayed a low antioxidant activity. In another study, ethyl acetate also extracted the lowest TPC and showed the lowest free radical scavenging activity in all plant samples that were analyzed [43].

Thus, the present findings indicate that polar solvents were obtaining more total phenolics and thus exhibited stronger antioxidant properties. These findings were in accordance with other studies [31,44,45]. Nevertheless, previous studies have also demonstrated the presence of a linear relationship between the antioxidant activity and phenolic content of plant extracts [46,47,48]. On the other hand, others have reported both the total phenolic and flavonoid contents to correlate with the antioxidant capacity of plants [49]. In fact, the same relationship could be implied for the presently investigated extracts, suggesting that the polyphenolic compounds are the main antioxidant components contributing to the high antioxidant ability, since methanolic and infusion extracts were richer in terms of TPC and TFC and showed higher antioxidant potency than the ethyl acetate extract.

Drugs acting as enzyme inhibitors make up a major part of pharmacy shelf. Likewise, drug development efforts at present are focused on identification and most of them involve the inhibition of enzymatic targets [50].

Neurodegenerative disorders, for example Alzheimer’s disease (AD), are often characterized by the degradation of neurotransmission. Accordingly, the aim of many treatment strategies includes the inhibition of acetylcholinesterase and thus the increasing level of acetylcholine in a synaptic gap [51]. Indeed, a huge array of cholinesterase inhibitors have been isolated from botanical sources, which have shown promising inhibitory activity against these enzymes [52,53].

In the current study, the ethyl acetate and methanol extracts were observed to be dual inhibitors of AChE and BChE. However, the methanolic extract showed higher inhibitory activity than the ethyl acetate extract. In contrast, the infusion extract inhibited AChE selectively (Table 4).

Tyrosinase is considered as the key enzyme in melanin synthesis process and it is used to treat hyperpigmentation problems. Recent studies have been shown that several natural compounds can be employed to inhibit tyrosinase [54,55].

In this study, a trend similar to the extracts’ cholinesterase inhibitory potential was seen for their anti-tyrosinase effect as well (MeOH > EA > Infusion). The methanolic extract was found to be the most potent tyrosinase inhibitor, followed by the ethyl acetate extract, whereas the least anti-tyrosinase capacity was demonstrated by the infusion extract (Table 4). Indeed, polyphenols are known to be good tyrosinase inhibitors [56]. For instance, ellagic acid was reported to prevent skin pigmentation resulting from UV irradiation by suppressing melanogenesis through the inhibition of tyrosinase activity. This inhibition is caused by chelation of the copper atoms on the tyrosinase molecules [57]. Additionally, Solimine, et al. [58] demonstrated in their study that polyphenol enriched fraction of rose oil distillation water strongly inhibited the tyrosinase with an IC_50_ of 0.41 μg/mL, while another fraction in which quercetin, kaempferol, and ellagic acid were identified showed anti-tyrosinase activity with IC_50_ values of 4.2 μM, 5.5 μM, and 5.2 μM, respectively, which is around 10 times more effective than that of kojic acid (56.1 μM) used as the positive control. On the other hand, docking simulations by [56] showed that polyphenols bind into the different sites of the tyrosinase and thus they could inhibit the ability of this enzyme.

Amylase and glucosidase are main enzyme in carbohydrate catabolism and they are keys to controlling blood glucose levels. Thus, the enzymes play pivotal roles in the management of diabetes mellitus [59]. To this end, synthetic compounds have been developed as inhibitors but most of them exhibited side effects. Thus, natural compounds are significant enzyme inhibitors instead of synthetic ones [60,61,62]. Interestingly herein, the ethyl acetate extract displayed the highest potency as an amylase inhibitor, followed by methanolic extract, whereas the extract obtained by infusion showed the least activity against amylase. The opposite was obtained for the anti-glucosidase inhibitory potential of the extracts, whereby only the infusion extract was found to inhibit glucosidase enzyme (6.82 ± 0.02 mmol ACAE/g), while ethyl acetate and methanol extracts demonstrated no anti-glucosidase effect (Table 4).

Indeed, natural products have been receiving increased interest over the past few decades for their effects as anticancer agents. In this study, *A. cordifolia* extracts were evaluated for their cytotoxicity against murine bone marrow cells (S7), murine melanoma cells (B16 4A5), and human hepatocellular carcinoma cells (HepG2). As shown in Table 5, the methanol and infusion extracts were found to be the most cytotoxic ones on murine bone marrow cells (S7) cells, compared to the ethyl acetate extract which was found to have low toxicity on S7 cells. For murine melanoma cells (B16 4A5), the cells showed reduced cell viability in the presence of ethyl acetate and infusion extracts, while the methanolic extract was relatively non-cytotoxic to B16 4A5 cells. On the other hand, the methanolic and infusion extracts exerted comparatively higher cytotoxicity on human hepatocellular carcinoma cells (HepG2), with selectivity values above 1, thus showing much lower cell viability (<20%) in contrast to the ethyl acetate extract. The ethyl acetate extract of *A. cordifolia* was cytotoxic on HepG2 cells, and together with the infusion extracts showed potent cytotoxicity on B16 4A5 cells (Table 5). Mitochondrial membrane potential (MMP) loss and increased ROS have been reported as a mode of apoptosis induction of plant extracts [63]. Interestingly, other *Alchornea* species such as *A. laxiflora* and especially *A. cordifolia* have been found to exert considerable cytotoxic properties against Leukemia CCRF-CEM cells [64].

**Figure 1:**
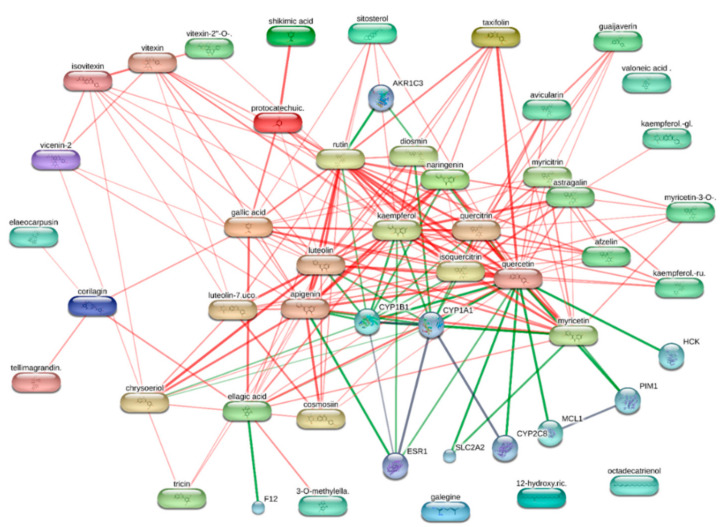
Components-targets analysis conducted on the phytochemicals identified by HPLC-MS in *A. cordifolia* methanol extract. Single protein targets predicted by STITCH platform (http://stitch.embl.de/) are shown. Quercetin, kaempferol, and apigenin were predicted to interact with estrogen receptor 1 (ESR1). Quercetin and myricetin showed a putative capacity to interact with pim-oncogene (PIM-1), whereas the sole quercetin was predicted to interact with hemopoietic cel kinase (HCK).

**Figure 2:**
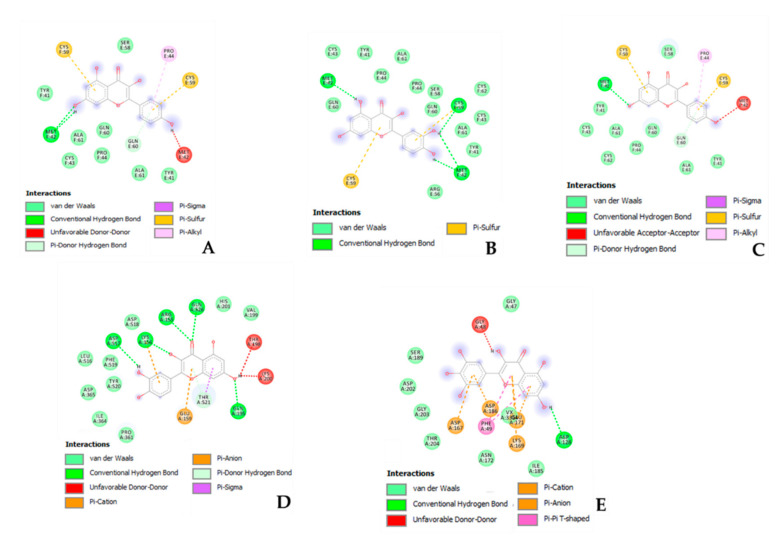
Putative interaction between kaempferol and estrogen receptor 1 (ESR1; PDB: 1HCQ); Free energy of binding (ΔG) and affinity (Ki) are −7.2 kcal/M and 5.4 µM, respectively (**A**). Putative interaction between apigenin and estrogen receptor 1 (ESR1; PDB: 1HCQ); Free energy of binding (ΔG) and affinity (Ki) are −7.3 kcal/M and 4.5 µM, respectively (**B**). Putative interaction between quercetin and estrogen receptor 1 (ESR1; PDB: 1HCQ); Free energy of binding (ΔG) and affinity (Ki) are −7.5 kcal/M and 3.2 µM, respectively (**C**). Putative interaction between quercetin and hemopoietic cell kinase (HCK; PDB: 1QCF); Free energy of binding (ΔG) and affinity (Ki) are −8.2 kcal/M and 1.0 µM, respectively (**D**). Putative interaction between myricetin and pim-1 oncogene (PIM-1; PDB: 3BGZ); Free energy of binding (ΔG) and affinity (Ki) are −7.3 kcal/M and 4.5 µM, respectively (**E**).

**Figure 3:**
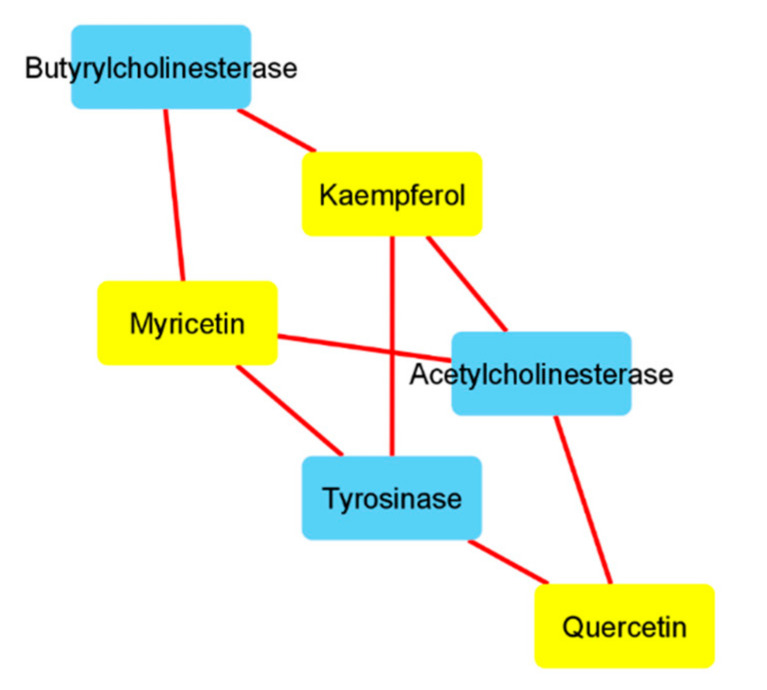
Components-targets analysis conducted on quercetin, kaempferol, and myricetin. The interactions between these phytochemicals and the enzymes tyrosinase and acethylcholinesterase were predicted by the bioinformatics platform SwissTargetPrediction (http://www.swisstargetprediction.ch/). Quercetin, kaempferol, and myricetin were selected according to their prominent position on the components-targets analysis (Figure 1), whereas the enzymes were selected based on the intrinsic enzyme inhibitory effects showed by the *A.cordifolia* extracts (Table 3).

**Figure 4:**
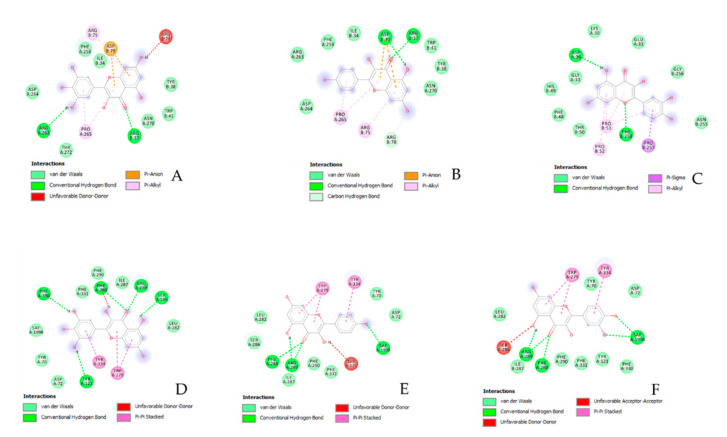
Putative interaction between myricetin and tyrosinase (PDB: 5I3B); Free energy of binding (ΔG) and affinity (Ki) are −7.2 kcal/M and 6.3 µM, respectively (**A**). Putative interaction between kaempferol and tyrosinase (PDB: 5I3B); Free energy of binding (ΔG) and affinity (Ki) are −7.1 kcal/M and 6.3 µM, respectively (**B**). Putative interaction between quercetin and tyrosinase (PDB: 5I3B); Free energy of binding (ΔG) and affinity (Ki) are −7.0 kcal/M and 7.6 µM, respectively (**C**). Putative interaction between myricetin and acethylcholinesterase (AchE; PDB: 1GQR); Free energy of binding (ΔG) and affinity (Ki) are −8.7 kcal/M and 0.4 µM, respectively (**D**). Putative interaction between kaempferol and acethylcholinesterase (AchE; PDB: 1GQR); Free energy of binding (ΔG) and affinity (Ki) are −8.0 kcal/M and 1.4 µM, respectively (**E**). Putative interaction between quercetin and acethylcholinesterase (AchE; PDB: 1GQR); Free energy of binding (ΔG) and affinity (Ki) are −8.7 kcal/M and 0.4 µM, respectively (**F**).

In order to explore the putative mechanisms underlying enzyme inhibition and anti-proliferative effects, components-targets analyses were conducted through the platforms STITCH and SwissTargetPrediction. Basically, the in silico study focused on the *A. cordifolia* methanol extract that showed the highest anti-tyrosinase and anti-cholinesterase activity. This extract also displayed an appreciable anti-proliferative effect against HepG2 cells. For the present components-targets, the defined structure, yielded by mass spectrometry analysis, was the elective criteria for the phytochemical selection. In this context, 40 phytochemicals, most being phenols and flavonoids, were run on the STITCH platform that showed putative interactions of several phytochemicals with enzymes, receptors, and oncogenes involved in cell metabolism and proliferation (Figure 1). It is of noteworthy interest that quercetin, apigenin, myricetin, and kaempferol were predicted to interact with pim-1 oncogene (PIM-1), estrogen receptor 1 (ESR1), and hemopoietic cell linase (HCK). These proteins have been proven to be useful for regulating tumor cell growth in preclinical models of cancer [65,66,67,68]. These putative interactions could mediate, at least partially, the observed anti-proliferative effects induced by *A. cordifolia* methanol extract. Additionally, docking experiments (Figure 2) and literature [69] suggest that binding interactions between selected phytochemicals and predicted proteins could occur at micromolar concentrations. The SwissTargetPrediction platform also highlighted putative interactions between kaempferol, myricetin, and apigenin towards tyrosinase and cholinesterases (Figure 3). Also in this case, literature data [58,69] and virtual screening experiments (Figure 4) showed good affinities of these phytochemicals towards tyrosinase and acethylcholinesterase (Ki: 0.4–7.4 µM). Collectively, these observations add to the aforementioned intrinsic enzyme inhibition (Table 3), by the *A. cordifolia* extracts. Nevertheless, we cannot exclude that the observed enzyme inhibition could partly depend on the total phenol and flavonoid content displayed by the extracts (Table 1). The literature data suggest phenol capability in inducing both scavenging/reducing and enzyme inhibitory effects [70,71]. Overall, the present in silico study supports future in vitro and in vivo investigations for confirming the present bio-pharmacological effects.

## 4. Conclusions

The findings from the present study indicate that *A. cordifolia* is an effective plant species that could be exploited for the management of oxidative stress related diseases, as well as act as a pharmacological agent against key illnesses (diabetes, neurodegeneration, skin hyperpigmentation, and cancer). Clearly, the active metabolites were more soluble in the highly polar solvents (methanol and water) than the less polar ethyl acetate and thus, they were able to yield higher phytochemical compounds. Moreover, the infusion and methanolic extracts showed higher antioxidant capacity, most likely due to their higher polyphenolic contents. Besides, the extracts were found to act as enzyme inhibitors and anticancer agents differentially. In this context, in silico experiments showed putative interactions of extract phytochemicals with several proteins and enzymes involved in cell metabolism and proliferation. It is also noteworthy to point out that the fact that the infusion extract of *A. cordifolia* was observed as a potent source of antioxidants and could also be used to manage other aforementioned diseases, which suggests its significance for its direct applications in the food and beverage industry as a natural supplement in health promotion. Nevertheless, advanced pharmacological and toxicological assessments (in vivo and clinical trials) need to be used to confirm efficacy and safety.

## Figures and Tables

**Table 1:** Total bioactive components of the tested extracts.

Extracts	Total Phenolic Content	Total Flavonoid Content
	(mg GAE/g Extract)	(mg RE/g Extract)
EA	120.38 ± 9.31 ^c^	9.66 ± 0.51 ^c^
MeOH	208.38 ± 0.41 ^b^	57.18 ± 0.94 ^a^
Infusion	213.12 ± 1.32 ^a^	46.30 ± 0.58 ^b^

Values expressed are means ± S.D. of three parallel measurements. GAE: Gallic acid equivalent; RE: Rutin equivalent. Different superscripts indicate significant differences in the extracts (*p* < 0.05).

**Table 2:** Chemical composition of *Alchornea cordifolia* extracts.

No.	Name	Formula	Rt	[M + H]^+^	[M − H]^−^	Ethyl Acetate	Methanol	Water
1	Shikimic acid	C7H10O5	1,27		17,304,500	+	+	+
2 ^1^	Gallic acid (3,4,5-Trihydroxybenzoic acid)	C7H6O5	2,60		16,901,370	+	+	+
3	Protocatechuic acid (3,4-Dihydroxybenzoic acid)	C7H6O4	5,43			+	+	+
4	Galegine (Isopentenyl guanidine)	C6H13N3	6,10	12,811,878		−	+	+
5	Unidentified alkaloid 1	C9H9NO5	8,98	21,205,590		−	+	−
6	Unidentified ellagic acid derivative	C21H10O13	11,31		46,900,432	+	+	+
7	Taxifolin-O-hexoside	C21H22O12	13,86		46,510,331	−	+	+
8	Putranjivain A	C46H36O31	15,71		108,311,623	+	+	+
9	Brevifolincarboxylic acid or isomer	C13H8O8	17,06		29,101,410	+	+	+
10	Procyanidin A isomer 1	C30H24O12	17,20		57,511,896	−	+	−
11	Potentillin or isomer	C41H28O26	17,30		93,507,906	+	+	+
12	Sanguisorbic acid dilactone	C21H10O13	17,36		46,900,432	−	+	−
13	Elaeocarpusin	C47H34O32	17,59		110,909,550	+	+	−
14	Procyanidin A isomer 2	C30H24O12	17,65		57,511,896	+	+	+
15	Valoneic acid dilactone	C21H10O13	17,70		46,900,432	+	+	+
16	Potentillin or isomer	C41H28O26	17,73		93,507,906	+	+	+
17	Corilagin or isomer	C27H22O18	17,89		63,307,279	+	+	+
18	Unidentified alkaloid 2	C13H10N2O3	18,07	24,206,914		−	+	−
19	Unidentified ellagic acid derivative	C21H10O13	18,53		46,900,432	+	+	+
20	Vicenin-2 (Apigenin-6,8-di-C-glucoside)	C27H30O15	19,33	59,516,630		−	+	+
21	Procyanidin A isomer 3	C30H24O12	19,45		57,511,896	+	+	−
22 ^1^	Taxifolin (Dihydroquercetin)	C15H12O7	19,84		30,305,048	+	+	+
23	Procyanidin A isomer 4	C30H24O12	20,18		57,511,896	+	+	+
24	Ellagic acid-4-O-glucoside	C20H16O13	20,37		46,305,127	−	+	+
25	Tellimagrandin I or isomer	C34H26O22	20,44		78,508,375	−	+	+
26	Quercetin-O-hexosylhexoside	C27H30O17	20,63		62,514,048	+	+	+
27	Myricetin-3’-O-glucoside	C21H20O13	21,36		47,908,257	+	+	+
28	Myricetin-O-rhamnosylhexoside isomer 1	C27H30O17	21,47		62,514,048	+	+	+
29	Unidentified hexahydroxydiphenoylhexose derivative	C34H26O22	21,61		78,508,375	+	+	+
30	Procyanidin A isomer 5	C30H24O12	21,68		57,511,896	+	+	−
31 ^1^	Vitexin (Apigenin-8-C-glucoside)	C21H20O10	21,79	43,311,347		+	+	+
32 ^1^	Vitexin-2’’-O-rhamnoside	C27H30O14	22,11	57,917,139		+	+	+
33	Taxifolin-O-pentoside	C20H20O11	22,37		43,509,274	+	+	+
34	Apigenin-C-hexoside-O-pentoside	C26H28O14	22,40	56,515,574		+	+	+
35	Myricitrin (Myricetin-3-O-rhamnoside)	C21H20O12	22,47		46,308,765	+	+	+
36	Isovitexin (Apigenin-6-C-glucoside)	C21H20O10	22,72	43,311,347		+	+	+
37	Luteolin-7-O-glucoside (Cynaroside)	C21H20O11	22,81		44,709,274	−	+	+
38	Luteolin-O-rhamnosylhexoside isomer 1	C27H30O15	22,84		59,315,065	+	+	+
39	Isovitexin-2’’-O-rhamnoside	C27H30O14	23,03	57,917,139		+	+	+
40	N1,N2-Diisopentenyl guanidine	C11H21N3	23,07	19,618,138		+	+	+
41	Hyperoside (Quercetin-3-O-galactoside)	C21H20O12	23,18		46,308,765	+	+	+
42	Ellagic acid-O-pentoside	C19H14O12	23,28		43,304,071	−	+	+
43 ^1^	Isoquercitrin (Quercetin-3-O-glucoside)	C21H20O12	23,40		46,308,765	+	+	+
44 ^1^	Rutin (Quercetin-3-O-rutinoside)	C27H30O16	23,46	61,116,122		+	+	+
45	Luteolin-O-rhamnosylhexoside isomer 2	C27H30O15	23,48		59,315,065	+	+	+
46	Eschweilenol C (Ellagic acid-4-O-rhamnoside)	C20H16O12	23,57		44,705,636	+	+	+
47	Reinutrin (Quercetin-3-O-xyloside)	C20H18O11	23,70		43,307,709	−	+	−
48	Ellagic acid	C14H6O8	23,84		30,099,845	+	+	+
49	Avicularin (Quercetin-3-O-arabinofuranoside)	C20H18O11	24,02		43,307,709	+	+	+
50	Mallotusinin or isomer	C41H26O25	24,20		91,706,850	+	+	+
51	Apigenin-O-rhamnosylhexoside isomer 1	C27H30O14	24,37		57,715,574	−	+	+
52 ^1^	Cosmosiin (Apigenin-7-O-glucoside)	C21H20O10	24,46	43,311,347		+	+	+
53	Myricetin-O-galloylrhamnoside	C28H24O16	24,57		61,509,861	+	+	+
54	Kaempferol-7-O-glucoside	C21H20O11	24,66		44,709,274	−	+	+
55 ^1^	Myricetin (3,3’,4’,5,5’,7-Hexahydroxyflavone)	C15H10O8	24,70		31,702,974	+	+	+
56	Chrysoeriol-O-hexoside	C22H22O11	24,73		46,110,839	+	+	+
57	Guaijaverin (Quercetin-3-O-arabinoside)	C20H18O11	24,74		43,307,709	+	+	+
58	Tricin-7-O-glucoside	C23H24O12	24,77		49,111,896	−	+	+
59	Apigenin-O-rhamnosylhexoside isomer 2	C27H30O14	24,89		57,715,574	−	+	+
60 ^1^	Diosmin (Diosmetin-7-O-rutinoside)	C28H32O15	24,96	60,918,195		+	+	+
61 ^1^	Quercitrin (Quercetin-3-O-rhamnoside)	C21H20O11	24,97		44,709,274	+	+	+
62	Astragalin (Kaempferol-3-O-glucoside)	C21H20O11	25,18		44,709,274	−	+	+
63	Unidentified ellagic acid derivative	C21H10O12	25,31		45,300,940	+	+	+
64	Kaempferol-3-O-rutinoside (Nicotiflorin)	C27H30O15	25,34		59,315,065	+	+	+
65	Kaempferol-O-pentoside	C20H18O10	25,40		41,708,218	+	+	+
66	3-O-Methylellagic acid	C15H8O8	26,26		31,501,410	+	+	+
67	Afzelin (Kaempferol-3-O-rhamnoside)	C21H20O10	26,92		43,109,782	+	+	+
68 ^1^	Quercetin (3,3’,4’,5,7-Pentahydroxyflavone)	C15H10O7	27,51		30,103,483	+	+	+
69 ^1^	Naringenin (4’,5,7-Trihydroxyflavanone)	C15H12O5	27,71		27,106,065	+	+	+
70 ^1^	Luteolin (3’,4’,5,7-Tetrahydroxyflavone)	C15H10O6	28,38		28,503,991	+	+	+
71	3,3’-Di-O-methylellagic acid	C16H10O8	28,45		32,902,975	+	+	+
72	Dihydroxy-methoxy(iso)flavone-O-hexoside	C22H22O10	28,59	44,712,913		+	+	+
73 ^1^	Kaempferol (3,4’,5,7-Tetrahydroxyflavone)	C15H10O6	29,87		28,503,991	+	+	+
74 ^1^	Apigenin (4’,5,7-Trihydroxyflavone)	C15H10O5	30,23		26,904,500	+	+	+
75 ^1^	Tricin (3’,5’-Dimethoxy-4’,5,7-trihydroxyflavone)	C17H14O7	30,41		32,906,613	+	+	+
76	Chrysoeriol (3’-Methoxy-4’,5,7-trihydroxyflavone)	C16H12O6	30,46		29,905,556	+	+	+
77	N1,N2,N3-Triisopentenyl guanidine	C16H29N3	30,82	26,424,398		+	+	+
78	3,3’,4-Tri-O-methylellagic acid	C17H12O8	30,84		34,304,540	+	+	+
79	3,3’,4,4’-Tetra-O-methylellagic acid	C18H14O8	32,67	35,907,670		+	+	+
80	Dihydroxy-methoxy(iso)flavone	C16H12O5	34,42	28,507,630		+	+	+
81	Octadecatrienol	C18H32O	45,71	26,525,314		+	+	−
82	2-Hydroxystearic acid	C18H36O3	47,01		29,925,863	+	+	−
83	β-Sitosterol	C29H50O	49,56	41,539,400		+	+	−
84	Myricetin-O-rhamnosylhexoside isomer 2	C27H30O17	22,02		62,514,048	−	−	+

^1^ Confirmed by standard. −: not detected; +: detected.

**Table 3:** Antioxidant activities of the tested samples.

Extracts	DPPH	ABTS	CUPRAC	FRAP	PPB	MCA
	(mg TE/g Extract)				(mmol TE/g)	(mg EDTAE/g)
EA	188.94 ± 0.15 ^c^	357.98 ± 0.76 ^c^	454.25 ± 7.69 ^c^	201.66 ± 6.00 ^c^	4.04 ± 0.10 ^c^	21.56 ± 0.55 ^b^
MeOH	500.38 ± 1.28 ^a^	900.64 ± 0.69 ^a^	1277.66 ± 2.98 ^b^	655.19 ± 16.00 ^b^	5.76 ± 0.51 ^b^	24.78 ± 1.18 ^a^
Infusion	490.94 ± 0.55 ^b^	839.30 ± 18.71 ^b^	1476.64 ± 1.08 ^a^	822.04 ± 6.54 ^a^	6.01 ± 0.10 ^a^	22.44 ± 0.82 ^b^

Values expressed are means ± S.D. of three parallel measurements. TE: Trolox equivalent; EDTAE: EDTA equivalent; PPB: phosphomolybdenum; MCA: metal chelating ability. Different superscripts indicate significant differences in the extracts (*p* < 0.05).

**Table 4:** Enzyme inhibitory properties of the tested extracts.

Extracts	AChE	BChE	Tyrosinase	α-Amylase	α-Glucosidase
	(mg GALAE/g)		(mg KAE/g)	(mmol ACAE/g)	
EA	4.47 ± 0.05 ^a^	5.81 ± 0.31 ^b^	119.11 ± 0.67 ^b^	1.19 ± 0.01 ^a^	na
MeOH	4.56 ± 0.06 ^a^	7.79 ± 0.21 ^a^	131.01 ± 0.84 ^a^	1.03 ± 0.06 ^b^	na
Infusion	2.02 ± 0.05 ^b^	na	59.53 ± 0.34 ^c^	0.21 ± 0.01 ^c^	6.82 ± 0.02

Values expressed are means ± S.D. of three parallel measurements. GALAE: Galantamine equivalent; KAE: Kojic acid equivalent; ACAE: Acarbose equivalent; na: not active. Different superscripts indicate significant differences in the extracts (*p* < 0.05).

**Table 5:** Cellular viability (%) of the extracts on HepG2, B16 4A5, and S17 cell lines applied at the concentration of 100 µg/mL.

Extracts	HepG2	SE	B16 4A5	SE	S17
DMSO 0.5%	101 ± 7 ^a^		88 ± 2 ^a^		79 ± 5 ^a^
EA	85 ± 4 ^b^	0.7	46 ± 1 ^c^	1.3	62 ± 3 ^b^
MeOH	17 ± 1 ^c^	1.2	88 ± 6 ^a^	0.2	21 ± 1 ^d^
Infusion	14 ± 1 ^d^	1.9	64 ± 1 ^b^	0.4	26 ± 1 ^c^

Values represent the mean ± standard error of the mean (SEM) of six replicates (*n* = 6). HepG_2_—human hepatocellular carcinoma cells; B16 4A5- murine melanoma cells; S17—murine bone marrow cells (non tumoral cells). In the same line, values marked by different letters were found to be significantly different according to the Tukey HSD test (*p* < 0.05). SE: Selectivity.

## Data Availability

The data presented in this study are available on request from the corresponding author.

## Supplementary Materials

The following are available online at https://www.mdpi.com/2218-273X/11/2/219/s1, Table S1: Chemical composition of ethyl acetate extract. Table S2: Chemical composition of methanol extract. Table S3: Chemical composition of water extract. Figure S1: Total ion chromatogram of ethyl acetate extract in positive ion mode. Figure S2: Total ion chromatogram of ethyl acetate extract in negative ion mode. Figure S3: Total ion chromatogram of methanol extract in positive ion mode. Figure S4: Total ion chromatogram of methanol extract in negative ion mode. Figure S5: Total ion chromatogram of water extract in positive ion mode. Figure S6. Total ion chromatogram of water extract in negative ion mode.
