# Supplementary material for: Deeper Insights on Alchornea cordifolia (Schumach. & Thonn.) Müll.Arg Extracts: Chemical Profiles, Biological Abilities, Network Analysis and Molecular Docking
Source: Biomolecules. 2021 Feb 4;11(2):219. doi: 10.3390/biom11020219 (PMC7913913; doi:10.3390/biom11020219)
Supplement: Supplementary file 1 [file biomolecules-11-00219-s001.pdf]

Article

Deeper Insights on *Alchornea cordifolia* (Schumach. & Thonn.) Müll.Arg Extracts: Chemical Profiles, Biological Abilities, Network Analysis and Molecular Docking

Kouadio Ibrahime Sinan [1], Gunes Ak [1], Ouattara Katinan Etienne [2], József Jekő [3], Zoltán Cziáky [3], Katalin Gupcsó [4], Maria João Rodrigues [5], Luisa Custodio [5], Mohamad Fawzi Mahomoodally [6], Jugreet B. Sharmeen [6], Luigi Brunetti [7], Sheila Leone [7], Lucia Recinella [7], Annalisa Chiavaroli [7], Giustino Orlando [7], Luigi Menghini [7], Massimo Tacchini [8,*], Claudio Ferrante [7,*] and Gokhan Zengin [1]

1 Physiology and Biochemistry Research Laboratory, Department of Biology, Science Faculty, Selcuk University, Campus, 42130 Konya, Turkey; sinankouadio@gmail.com (K.I.S.); akguneselcuk@gmail.com (G.A.); gokhanzengin@selcuk.edu.tr (G.Z.)
2 Laboratoire de Botanique, UFR Biosciences, Université Félix Houphouët-Boigny, 01Abidjan, Ivory Coast; katinan.etienne@gmail.com
3 Agricultural and Molecular Research and Service Institute, University of Nyíregyháza, 4400, Nyíregyháza, Hungary; jjozsi@gmail.com (J.J.); cziaky.zoltan@nye.hu (Z.C.)
4 Sotiva Seed Ltd., H-4440 Tiszavasvári, Petőfi str.Hungary; sotiva@sotiva.hu
5 Centre of Marine Sciences, University of Algarve, Faculty of Sciences and Technology, Ed. 7, Campus of Gambelas, 8005-139 Faro, Portugal; mary_p@sapo.pt (M.J.R.); lcustodio@ualg.pt (L.C.)
6 Department of Health Sciences, Faculty of Medicine and Health Sciences, University of Mauritius, Réduit, Mauritius; f.mahomoodally@uom.ac.mu (M.F.M.); sharmeenjugs@gmail.com (J.B.S.)
7 Department of Pharmacy, "G. d'Annunzio University", via dei Vestini n. 31, 66100 Chieti, Italy; luigi.brunetti@unich.it (L.B.); sheila.leone@unich.it (S.L.); lucia.recinella@unich.it (L.R.); annalisa.chiavaroli@unich.it (A.C.); giustino.orlando@unich.it (G.O.); luigi.menghini@unich.it (L.M.)
8 Department of Life Sciences and Biotechnology (SVeB), UR7 Terra&Acqua Tech, University of Ferrara, 44121 Ferrara, Italy
* Correspondence: claudio.ferrante@unich.it (C.F.); massimo.tacchini@unife.it (M.T.)

Assays for Total Phenolic and Flavonoid Contents

The total phenolic content was determined by employing the methods given in the literature with some modification. Sample solution (0.25 mL) was mixed with diluted Folin–Ciocalteu reagent (1 mL, 1:9, v/v) and shaken vigorously. After 3 min, Na_2CO_3 solution (0.75 mL, 1%) was added and the sample absorbance was read at 760 nm after a 2 h incubation at room temperature. The total phenolic content was expressed as milligrams of gallic acid equivalents (mg GAE/g extract)[1].

The total flavonoid content was determined using the $AlCl_3$ method. Briefly, sample solution (1 mL) was mixed with the same volume of aluminum trichloride (2%) in methanol. Similarly, a blank was prepared by adding sample solution (1 mL) to methanol (1 mL) without $AlCl_3$. The sample and blank absorbances were read at 415 nm after a 10 min incubation at room temperature. The absorbance of the blank was subtracted from that of the sample. Rutin was used as a reference standard and the total flavonoid content was expressed as milligrams of rutin equivalents (mg RE/g extract) [1].

Determination of Antioxidant and Enzyme Inhibitory Effects

Antioxidant (DPPH and ABTS radical scavenging, reducing power (CUPRAC and FRAP), phosphomolybdenum and metal chelating (ferrozine method)) and enzyme inhibitory activities (cholinesterase (Elmann's method), tyrosinase (dopachrome method), α-amylase (iodine/potassium iodide method), α -glucosidase (chromogenic PNPG method) and pancreatic lipase (*p*-nitrophenyl butyrate (p-NPB) method) were determined using the methods previously described by Uysal et al. [1] and Grochowski et al. [2]

For the DPPH (1,1-diphenyl-2-picrylhydrazyl) radical scavenging assay: Sample solution was added to 4 mL of a 0.004% methanol solution of DPPH. The sample absorbance was read at 517 nm after a 30 min incubation at room temperature in the dark. DPPH radical scavenging activity was expressed as milligrams of trolox equivalents (mg TE/g extract).

For ABTS (2,2′-azino-bis(3-ethylbenzothiazoline) 6-sulfonic acid) radical scavenging assay: Briefly, ABTS+ was produced directly by reacting 7 mM ABTS solution with 2.45 mM potassium persulfate and allowing the mixture to stand for 12–16 in the dark at room temperature. Prior to beginning the assay, ABTS solution was diluted with methanol to an absorbance of 0.700 ± 0.02 at 734 nm. Sample solution was added to ABTS solution (2 mL) and mixed. The sample absorbance was read at 734 nm after a 30 min incubation at room temperature. The ABTS radical scavenging activity was expressed as milligrams of trolox equivalents (mg TE/g extract).

For CUPRAC (cupric ion reducing activity) activity assay: Sample solution was added to premixed reaction mixture containing $CuCl_2$ (1 mL, 10 mM), neocuproine (1 mL, 7.5 mM) and NH_4Ac buffer (1 mL, 1 M, pH 7.0). Similarly, a blank was prepared by adding sample solution (0.5 mL) to premixed reaction mixture (3 mL) without $CuCl_2$. Then, the sample and blank absorbances were read at 450 nm after a 30 min incubation at room

temperature. The absorbance of the blank was subtracted from that of the sample. CUPRAC activity was expressed as milligrams of trolox equivalents (mg TE/g extract).

For FRAP (ferric reducing antioxidant power) activity assay: Sample solution was added to premixed FRAP reagent (2 mL) containing acetate buffer (0.3 M, pH 3.6), 2,4,6-tris(2-pyridyl)-S-triazine (TPTZ) (10 mM) in 40 mM HCl and ferric chloride (20 mM) in a ratio of 10:1:1 (v/v/v). Then, the sample absorbance was read at 593 nm after a 30 min incubation at room temperature. FRAP activity was expressed as milligrams of trolox equivalents (mg TE/g extract).

For phosphomolybdenum method: Sample solution was combined with 3 mL of reagent solution (0.6 M sulfuric acid, 28 mM sodium phosphate and 4 mM ammonium molybdate). The sample absorbance was read at 695 nm after a 90 min incubation at 95 °C. The total antioxidant capacity was expressed as millimoles of trolox equivalents (mmol TE/g extract).

For metal chelating activity assay: Briefly, sample solution was added to $FeCl_2$ solution (0.05 mL, 2 mM). The reaction was initiated by the addition of 5 mM ferrozine (0.2 mL). Similarly, a blank was prepared by adding sample solution (2 mL) to $FeCl_2$ solution (0.05 mL, 2 mM) and water (0.2 mL) without ferrozine. Then, the sample and blank absorbances were read at 562 nm after 10 min incubation at room temperature. The absorbance of the blank was sub-tracted from that of the sample. The metal chelating activity was expressed as milligrams of EDTA (disodium edetate) equivalents (mg EDTAE/g extract).

For Cholinesterase (ChE) inhibitory activity assay: Sample solution (was mixed with DTNB (5,5-dithio-bis(2-nitrobenzoic) acid, Sigma, St. Louis, MO, USA) (125 µL) and AChE (acetylcholines-terase (Electric ell acetylcholinesterase, Type-VI-S, EC 3.1.1.7,Sigma)), or BChE (butyrylcholinesterase (horse serum butyrylcholinesterase, EC 3.1.1.8, Sigma)) solution (25 µL) in Tris–HCl buffer (pH 8.0) in a 96-well microplate and incubated for 15 min at 25 °C. The reaction was then initiated with the addition of acetylthiocholine iodide (ATCI, Sigma) or butyrylthiocholine chloride (BTCl, Sigma) (25 µL). Similarly, a blank was prepared by adding sample solution to all reaction reagents without enzyme (AChE or BChE) solution. The sample and blank absorbances were read at 405 nm after 10 min incubation at 25 °C. The absorbance of the blank was subtracted from that of the sample and the cholinesterase inhibitory activity was expressed as galanthamine equivalents (mgGALAE/g extract).

For Tyrosinase inhibitory activity assay: Sample solution was mixed with tyrosinase solution (40 μL, Sigma) and phosphate buffer (100 μL, pH 6.8) in a 96-well microplate and incubated for 15 min at 25 °C. The reaction was then initiated with the addition of L-DOPA (40 μL, Sigma). Similarly, a blank was prepared by adding sample solution to all reaction reagents without enzyme (tyrosinase) solution. The sample and blank absorbances were read at 492 nm after a 10 min incubation at 25 °C. The absorbance of the blank was subtracted from that of the sample and the tyrosinase inhibitory activity was expressed as kojic acid equivalents (mgKAE/g extract).

For α-amylase inhibitory activity assay: Sample solution was mixed with α-amylase solution (ex-porcine pancreas, EC 3.2.1.1, Sigma) (50 μL) in phosphate buffer (pH 6.9 with 6 mM sodium chloride) in a 96-well microplate and incubated for 10 min at 37 °C. After pre-incubation, the reaction was initiated with the addition of starch solution (50 μL, 0.05%). Similarly, a blank was prepared by adding sample solution to all reaction reagents without enzyme (α-amylase) solution. The reaction mixture was incubated 10 min at 37 °C. The reaction was then stopped with the addition of HCl (25 μL, 1 M). This was followed by addition of the iodine-potassium iodide solution (100 μL). The sample and blank absorbances were read at 630 nm. The absorbance of the blank was subtracted from that of the sample and the α-amylase inhibitory activity was expressed as acarbose equivalents (mmol ACE/g extract).

For α-glucosidase inhibitory activity assay: Sample solution was mixed with glutathione (50 μL), α-glucosidase solution (from Saccharomyces cerevisiae, EC 3.2.1.20, Sigma) (50 μL) in phosphate buffer (pH 6.8) and PNPG (4-N-trophenyl-α-D-glucopyranoside, Sigma) (50 μL) in a 96-well microplate and incubated for 15 min at 37 °C. Similarly, a blank was prepared by adding sample solution to all reaction reagents without enzyme (α-glucosidase) solution. The reaction was then stopped with the addition of sodium carbonate (50 μL, 0.2 M). The sample and blank absorbances were read at 400 nm. The absorbance of the blank was subtracted from that of the sample and the α-glucosidase inhibitory activity was expressed as acarbose equivalents (mmol ACE/g extract).

Table S1. Chemical composition of ethyl acetate extract.

No.	Name	Formula	Rt	[M + H]+	[M - H]-	Fragment 1	Fragment 2	Fragment 3	Fragment 4	Fragment 5	Literature
1	Shikimic acid	C7H10O5	1,42		173,04500	155,0338	137,0232	111,0437	93,0330	73,0279	[3]
2[1]	Gallic acid (3,4,5-Trihydroxybenzoic acid)	C7H6O5	2,64		169,01370	125,0231	97,0281	81,0332	79,0176	69,0330	[3]
3	Protocatechuic acid (3,4-Dihydroxybenzoic acid)	C7H6O4	5,49		153,01879	109,0281	108,0203	91,0173	81,0330		[3]
4	Putranjivain A	C46H36O31	15,73		1083,11623	935,0770	853,0719	633,0751	463,0521	300,9992	[4]
5	Brevifolincarboxylic acid or isomer	C13H8O8	17,09		291,01410	247,0247	219,0294	203,0344	191,0343	175,0390	
6	Potentillin or isomer	C41H28O26	17,31		935,07906	765,0576	463,0522	300,9991	299,9914	275,0201	
7	Elaeocarpusin	C47H34O32	17,58		1109,09550	1049,0756	935,0811	633,0718	463,0520	300,9992	[4]
8	Procyanidin A isomer 2	C30H24O12	17,66		575,11896	539,0963	449,0858	407,0780	285,0406	125,0230	
9	Valoneic acid dilactone	C21H10O13	17,71		469,00432	450,9931	425,0160	407,0026	300,9992	299,9913	
10	Potentillin or isomer	C41H28O26	17,73		935,07906	**765**,0558	463,0524	300,9992	299,9908	275,0196	
11	Corilagin or isomer	C27H22O18	17,92		633,07279	463,0523	419,0623	300,9992	275,0200	169,0132	
12	Unidentified ellagic acid derivative	C21H10O13	18,53		469,00432	425,0159	300,9994	299,9914			
13	Procyanidin A isomer 3	C30H24O12	19,46		575,11896	539,0999	449,0876	407,0777	285,0411	125,0231	
14[1]	Taxifolin (Dihydroquercetin)	C15H12O7	19,85		303,05048	285,0408	217,0505	175,0391	153,0181	125,0230	
15	Procyanidin A isomer 4	C30H24O12	20,19		575,11896	539,0995	449,0884	407,0782	285,0408	125,0230	
16	Quercetin-O-hexosylhexoside	C27H30O17	20,64		625,14048	301,0357	300,0280	271,0250	255,0299	178,9977	
17	Myricetin-3'-O-glucoside	C21H20O13	21,39		479,08257	317,0306	316,0226	287,0202	271,0251	242,0217	
18	Myricetin-O-rhamnosylhexoside isomer 1	C27H30O17	21,51		625,14048	317,0310	316,0226	287,0201	271,0252	178,9979	
19	Unidentified hexahydroxydiphenoylhexose derivative	C34H26O22	21,63		785,08375	615,0637	463,0502	300,9992	275,0200	169,0132	

20	Procyanidin A isomer 5	C30H24O12	21,70	575,11896	539,0986	449,0869	407,0784	285,0409	125,0231	
21[1]	Vitexin (Apigenin-8-C-glucoside)	C21H20O10	21,82	433,11347	415,1032	397,0922	379,0817	313,0712	283,0606	
22[1]	Vitexin-2''-O-rhamnoside	C27H30O14	22,11	579,17139	433,1137	415,1031	313,0710	283,0605	271,0605	
23	Taxifolin-O-pentoside	C20H20O11	22,38	435,09274	303,0515	285,0407	178,9977	151,0025	125,0230	
24	Apigenin-C-hexoside-O-pentoside	C26H28O14	22,43	565,15574	433,1134	415,1030	313,0710	283,0605	271,0605	
25	Myricitrin (Myricetin-3-O-rhamnoside)	C21H20O12	22,51	463,08765	317,0305	316,0225	287,0201	271,0251	178,9975	
26	Isovitexin (Apigenin-6-C-glucoside)	C21H20O10	22,73	433,11347	415,1031	397,0926	379,0815	313,0711	283,0605	
27	Luteolin-O-rhamnosylhexoside isomer 1	C27H30O15	22,86	593,15065	447,0927	285,0406	284,0331	151,0024	133,0281	
28	Isovitexin-2''-O-rhamnoside	C27H30O14	23,05	579,17139	433,1137	415,1028	313,0710	283,0605	271,0605	
29	N1,N2-Diisopentenyl guanidine	C11H21N3	23,06	196,18138	128,1186	69,0706	60,0564			[5]
30	Hyperoside (Quercetin-3-O-galactoside)	C21H20O12	23,19	463,08765	301,0356	300,0277	271,0249	255,0297	178,9977	[6]
31[1]	Isoquercitrin (Quercetin-3-O-glucoside)	C21H20O12	23,40	463,08765	301,0356	300,0277	271,0249	255,0298	178,9977	
32[1]	Rutin (Quercetin-3-O-rutinoside)	C27H30O16	23,48	611,16122	465,1032	303,0503	129,0552	85,0291	71,0499	
33	Luteolin-O-rhamnosylhexoside isomer 2	C27H30O15	23,49	593,15065	447,0935	285,0407	284,0329	151,0024	133,0284	
34	Eschweilenol C (Ellagic acid-4-O-rhamnoside)	C20H16O12	23,60	447,05636	300,9987	299,9910	283,9985	257,0084		
35	Ellagic acid	C14H6O8	23,91	300,99845	283,9964	257,0088	229,0138	201,0188	185,0238	[3]
36	Avicularin (Quercetin-3-O-arabinofuranoside)	C20H18O11	24,02	433,07709	301,0356	300,0277	271,0249	255,0297	178,9976	[6]
37	Mallotusinin or isomer	C41H26O25	24,20	917,06850	747,0488	615,0615	445,0403	300,9992	275,0200	
38[1]	Cosmosiin (Apigenin-7-O-glucoside)	C21H20O10	24,47	433,11347	271,0602	153,0182	145,0284	119,0496		

39	Myricetin-O-galloylrhamnoside	C28H24O16	24,57	615,09861	463,0884	317,0306	316,0226	271,0251	169,0132	
40[1]	Myricetin (3,3',4',5,5',7-Hexahydroxyflavone)	C15H10O8	24,71	317,02974	271,0252	178,9976	165,0179	151,0024	137,0232	
41	Chrysoeriol-O-hexoside	C22H22O11	24,73	461,10839	446,0858	299,0563	298,0487	297,0404	283,0250	
42	Guaijaverin (Quercetin-3-O-arabinoside)	C20H18O11	24,74	433,07709	301,0354	300,0277	271,0250	255,0297	178,9975	[6]
43[1]	Quercitrin (Quercetin-3-O-rhamnoside)	C21H20O11	24,96	447,09274	301,0356	300,0277	271,0249	255,0297	178,9976	
44[1]	Diosmin (Diosmetin-7-O-rutinoside)	C28H32O15	24,98	609,18195	463,1239	301,0709	286,0475	129,0550	85,0290	
45	Kaempferol-3-O-rutinoside (Nicotiflorin)	C27H30O15	25,34	593,15065	327,0513	285,0407	284,0329	255,0298	227,0343	
46	Kaempferol-O-pentoside	C20H18O10	25,40	417,08218	285,0408	284,0330	255,0298	227,0345	151,0023	
47	3-O-Methylellagic acid	C15H8O8	26,24	315,01410	299,9913	244,0004	228,0062	200,0102		
48	Afzelin (Kaempferol-3-O-rhamnoside)	C21H20O10	26,91	431,09782	285,0409	284,0331	255,0299	227,0346	151,0025	
49[1]	Quercetin (3,3',4',5,7-Pentahydroxyflavone)	C15H10O7	27,51	301,03483	273,0407	178,9977	151,0025	121,0281	107,0125	[6]
50[1]	Naringenin (4',5,7-Trihydroxyflavanone)	C15H12O5	27,72	271,06065	227,0703	177,0183	151,0025	119,0488	107,0124	
51[1]	Luteolin (3',4',5,7-Tetrahydroxyflavone)	C15H10O6	28,37	285,03991	217,0496	199,0395	175,0391	151,0025	133,0282	
52	3,3'-Di-O-methylellagic acid	C16H10O8	28,43	329,02975	314,0074	312,9996	298,9837	270,9887		
53	Dihydroxy-methoxy(iso)flavone-O-hexoside	C22H22O10	28,57	447,12913	285,0762	270,0527	242,0576	153,0185		
54[1]	Kaempferol (3,4',5,7-Tetrahydroxyflavone)	C15H10O6	29,87	285,03991	257,0446	229,0497	213,0552	151,0025	107,0125	
55[1]	Apigenin (4',5,7-Trihydroxyflavone)	C15H10O5	30,22	269,04500	225,0552	151,0024	149,0232	117,0331	107,0125	
56[1]	Tricin (3',5'-Dimethoxy-4',5,7-trihydroxyflavone)	C17H14O7	30,39	329,06613	314,0436	313,0360	299,0199	271,0248	227,0345	
57	Chrysoeriol (3'-Methoxy-4',5,7-trihydroxyflavone)	C16H12O6	30,44	299,05556	284,0329	256,0376	227,0348	151,0026	107,0128	
58	3,3',4-Tri-O-methylellagic acid	C17H12O8	30,81	343,04540	328,0227	312,9993	297,9757	285,0044	269,9809	
59	N1,N2,N3-Triisopentenyl guanidine	C16H29N3	30,87	264,24398	196,1813	128,1187	69,0706	60,0565		[5]

60	3,3',4,4'-Tetra-O-methylellagic acid	$C_{18}H_{14}O_8$	32,63	359,07670	344,0531	343,0458	329,0295	313,0346	285,0397	
61	Dihydroxy-methoxy(iso)flavone	$C_{16}H_{12}O_5$	34,41	285,07630	270,0526	242,0575	153,0179	133,0657		
62	Octadecatrienol	$C_{18}H_{32}O$	45,64	265,25314	247,2424	191,1800	95,0862	83,0862	69,0706	
63	2-Hydroxystearic acid	$C_{18}H_{36}O_3$	46,99	299,25863	281,2482	253,2536	251,2377	225,2212		
64	β-Sitosterol	$C_{29}H_{50}O$	49,55	415,39400	397,3835	161,1323	135,1171	95,0860	81,0706	[7]

Table S2. Chemical composition of methanol extract.

No.	Name	Formula	Rt	[M + H]+	[M - H]-	Fragment 1	Fragment 2	Fragment 3	Fragment 4	Fragment 5	Literature
1	Shikimic acid	$C_7H_{10}O_5$	1,27		173,04500	155,0338	137,0232	111,0437	93,0330	73,0279	
2[1]	Gallic acid (3,4,5-Trihydroxybenzoic acid)	$C_7H_6O_5$	2,60		169,01370	125,0230	97,0281	81,0332	79,0175	69,0330	[3]
3	Protocatechuic acid (3,4-Dihydroxybenzoic acid)	$C_7H_6O_4$	5,43		153,01879	109,0281	108,0202	91,0175	81,0330		[3]
4	Galegine (Isopentenyl guanidine)	$C_6H_{13}N_3$	6,10	128,11878		69,0706	60,0565				
5	Unidentified alkaloid 1	$C_9H_9NO_5$	8,98	212,05590		194,0453	184,0608	166,0501			
6	Unidentified ellagic acid derivative	$C_{21}H_{10}O_{13}$	11,31		469,00432	450,9945	425,0154	407,0046	379,0096	299,9914	
7	Taxifolin-O-hexoside	$C_{21}H_{22}O_{12}$	13,86		465,10331	303,0512	285,0405	178,9976	151,0023	125,0229	
8	Putranjivain A	$C_{46}H_{36}O_{31}$	15,71		1083,11623	935,0820	853,0719	633,0713	463,0503	300,9992	[4]
9	Brevifolincarboxylic acid or isomer	$C_{13}H_8O_8$	17,06		291,01410	247,0246	219,0293	203,0344	191,0342	175,0391	
10	Procyanidin A isomer 1	$C_{30}H_{24}O_{12}$	17,20		575,11896	539,0986	449,0897	407,0768	285,0410	125,0231	
11	Potentillin or isomer	$C_{41}H_{28}O_{26}$	17,30		935,07906	765,0581	463,0525	300,9991	299,9913	275,0200	
12	Sanguisorbic acid dilactone	$C_{21}H_{10}O_{13}$	17,36		469,00432	450,9939	425,0167	407,0040	300,9991	299,9914	
13	Elaeocarpusin	$C_{47}H_{34}O_{32}$	17,59		1109,09550	1049,0724	935,0795	633,0729	463,0519	300,9992	[4]
14	Procyanidin A isomer 2	$C_{30}H_{24}O_{12}$	17,65		575,11896	539,0982	449,0873	407,0782	285,0405	125,0228	
15	Valoneic acid dilactone	$C_{21}H_{10}O_{13}$	17,70		469,00432	450,9923	425,0151	407,0026	300,9991	299,9914	
16	Potentillin or isomer	$C_{41}H_{28}O_{26}$	17,73		935,07906	765,0609	463,0512	300,9991	299,9913	275,0201	
17	Corilagin or isomer	$C_{27}H_{22}O_{18}$	17,89		633,07279	463,0516	419,0624	300,9992	275,0201	169,0126	
18	Unidentified alkaloid 2	$C_{13}H_{10}N_2O_3$	18,07	242,06914		215,0818	197,0713	169,0762			
19	Unidentified ellagic acid derivative	$C_{21}H_{10}O_{13}$	18,53		469,00432	425,0157	300,9991	299,9913			
20	Vicenin-2 (Apigenin-6,8-di-C-glucoside)	$C_{27}H_{30}O_{15}$	19,33	595,16630		577,1566	559,1458	457,1137	325,0710	295,0606	
21	Procyanidin A isomer 3	$C_{30}H_{24}O_{12}$	19,45		575,11896	539,0994	449,0872	407,0744	285,0406	125,0229	

22[1]	Taxifolin (Dihydroquercetin)	C15H12O7	19,84		303,05048	285,0411	217,0500	175,0389	153,0176	125,0230	
23	Procyanidin A isomer 4	C30H24O12	20,18		575,11896	539,1018	449,0894	407,0767	285,0407	125,0231	
24	Ellagic acid-4-O-glucoside	C20H16O13	20,37		463,05127	300,9991	299,9913	298,9843	283,9971	257,0071	
25	Tellimagrandin I or isomer	C34H26O22	20,44		785,08375	633,0740	463,0516	300,9992	275,0200	169,0132	
26	Quercetin-O-hexosylhexoside	C27H30O17	20,63		625,14048	301,0356	300,0278	271,0251	255,0295	178,9974	
27	Myricetin-3'-O-glucoside	C21H20O13	21,36		479,08257	317,0304	316,0225	287,0201	271,0249	242,0219	
28	Myricetin-O-rhamnosylhexoside isomer 1	C27H30O17	21,47		625,14048	317,0301	316,0226	287,0207	271,0249	178,9982	
29	Unidentified hexahydroxydiphenoylhexose derivative	C34H26O22	21,61		785,08375	615,0622	463,0522	300,9991	275,0201	169,0131	
30	Procyanidin A isomer 5	C30H24O12	21,68		575,11896	539,0992	449,0890	407,0774	285,0411	125,0231	
31[1]	Vitexin (Apigenin-8-C-glucoside)	C21H20O10	21,79	433,11347		415,1030	397,0924	379,0817	313,0710	283,0604	
32[1]	Vitexin-2''-O-rhamnoside	C27H30O14	22,11	579,17139		433,1134	415,1030	313,0710	283,0604	271,0604	
33	Taxifolin-O-pentoside	C20H20O11	22,37		435,09274	303,0513	285,0408	178,9977	151,0025	125,0230	
34	Apigenin-C-hexoside-O-pentoside	C26H28O14	22,40	565,15574		433,1134	415,1029	313,0709	283,0605	271,0612	
35	Myricitrin (Myricetin-3-O-rhamnoside)	C21H20O12	22,47		463,08765	317,0303	316,0226	287,0202	271,0249	178,9976	
36	Isovitexin (Apigenin-6-C-glucoside)	C21H20O10	22,72	433,11347		415,1024	397,0923	379,0815	313,0710	283,0604	
37	Luteolin-7-O-glucoside (Cynaroside)	C21H20O11	22,81		447,09274	327,0507	285,0407	284,0328	256,0377	133,0280	
38	Luteolin-O-rhamnosylhexoside isomer 1	C27H30O15	22,84		593,15065	447,0956	285,0407	284,0329	151,0026	133,0284	
39	Isovitexin-2''-O-rhamnoside	C27H30O14	23,03	579,17139		433,1135	415,1029	313,0710	283,0604	271,0603	
40	N1,N2-Diisopentenyl guanidine	C11H21N3	23,07	196,18138		128,1187	69,0706	60,0565			[5]
41	Hyperoside (Quercetin-3-O-galactoside)	C21H20O12	23,18		463,08765	301,0359	300,0278	271,0250	255,0299	178,9977	[6]
42	Ellagic acid-O-pentoside	C19H14O12	23,28		433,04071	300,9992	299,9914	283,9970	257,0095		
43[1]	Isoquercitrin (Quercetin-3-O-glucoside)	C21H20O12	23,40		463,08765	301,0357	300,0278	271,0251	255,0299	178,9977	
44[1]	Rutin (Quercetin-3-O-rutinoside)	C27H30O16	23,46	611,16122		465,1038	303,0502	129,0550	85,0290	71,0498	

45	Luteolin-O-rhamnosylhexoside isomer 2	C27H30O15	23,48		593,15065	447,0928	285,0407	284,0330	151,0022	133,0277	
46	Eschweilenol C (Ellagic acid-4-O-rhamnoside)	C20H16O12	23,57		447,05636	300,9991	299,9912	283,9985	257,0092		
47	Reinutrin (Quercetin-3-O-xyloside)	C20H18O11	23,70		433,07709	301,0355	300,0277	271,0252	255,0297	178,9972	
48	Ellagic acid	C14H6O8	23,84		300,99845	283,9967	257,0085	229,0137	201,0189	185,0237	[3]
49	Avicularin (Quercetin-3-O-arabinofuranoside)	C20H18O11	24,02		433,07709	301,0356	300,0278	271,0250	255,0298	178,9980	[6]
50	Mallotusinin or isomer	C41H26O25	24,20		917,06850	747,0504	615,0609	445,0416	300,9991	275,0201	
51	Apigenin-O-rhamnosylhexoside isomer 1	C27H30O14	24,37		577,15574	269,0457	268,0370	151,0030	117,0334		
52[1]	Cosmosiin (Apigenin-7-O-glucoside)	C21H20O10	24,46	433,11347		271,0602	153,0185	145,0284	119,0494		
53	Myricetin-O-galloylrhamnoside	C28H24O16	24,57		615,09861	463,0886	317,0303	316,0225	271,0249	169,0132	
54	Kaempferol-7-O-glucoside	C21H20O11	24,66		447,09274	285,0407	284,0329	255,0298	227,0345	151,0023	
55[1]	Myricetin (3,3',4',5,5',7-Hexahydroxyflavone)	C15H10O8	24,70		317,02974	271,0234	178,9977	165,0182	151,0025	137,0232	
56	Chrysoeriol-O-hexoside	C22H22O11	24,73		461,10839	446,0860	299,0561	298,0487	297,0415	283,0251	
57	Guaijaverin (Quercetin-3-O-arabinoside)	C20H18O11	24,74		433,07709	301,0353	300,0274	271,0249	255,0297	178,9977	[6]
58	Tricin-7-O-glucoside	C23H24O12	24,77		491,11896	476,0966	461,0730	328,0587	313,0357	285,0404	
59	Apigenin-O-rhamnosylhexoside isomer 2	C27H30O14	24,89		577,15574	413,0876	269,0456	268,0378	151,0030	117,0333	
60[1]	Diosmin (Diosmetin-7-O-rutinoside)	C28H32O15	24,96	609,18195		463,1238	301,0707	286,0474	129,0548	85,0290	
61[1]	Quercitrin (Quercetin-3-O-rhamnoside)	C21H20O11	24,97		447,09274	301,0355	300,0277	271,0249	255,0299	178,9976	
62	Astragalin (Kaempferol-3-O-glucoside)	C21H20O11	25,18		447,09274	285,0407	284,0329	255,0298	227,0345	151,0025	
63	Unidentified ellagic acid derivative	C21H10O12	25,31		453,00940	434,9998	409,0201	391,0108	367,0086	300,9993	
64	Kaempferol-3-O-rutinoside (Nicotiflorin)	C27H30O15	25,34		593,15065	327,0490	285,0407	284,0329	255,0298	227,0342	
65	Kaempferol-O-pentoside	C20H18O10	25,40		417,08218	285,0407	284,0329	255,0298	227,0345	151,0026	
66	3-O-Methylellagic acid	C15H8O8	26,26		315,01410	299,9913	244,0013	228,0062	200,0106		
67	Afzelin (Kaempferol-3-O-rhamnoside)	C21H20O10	26,92		431,09782	285,0407	284,0330	255,0298	227,0345	151,0027	

68[1]	Quercetin (3,3',4',5,7-Pentahydroxyflavone)	C15H10O7	27,51		301,03483	273,0411	178,9977	151,0025	121,0281	107,0125	[6]
69[1]	Naringenin (4',5,7-Trihydroxyflavanone)	C15H12O5	27,71		271,06065	227,0711	177,0183	151,0025	119,0488	107,0125	
70[1]	Luteolin (3',4',5,7-Tetrahydroxyflavone)	C15H10O6	28,38		285,03991	217,0494	199,0391	175,0390	151,0025	133,0282	
71	3,3'-Di-O-methylellagic acid	C16H10O8	28,45		329,02975	314,0073	312,9993	298,9836	270,9887		
72	Dihydroxy-methoxy(iso)flavone-O-hexoside	C22H22O10	28,59	447,12913		285,0761	270,0524	242,0572	153,0167		
73[1]	Kaempferol (3,4',5,7-Tetrahydroxyflavone)	C15H10O6	29,87		285,03991	257,0456	229,0502	213,0549	151,0022	107,0121	
74[1]	Apigenin (4',5,7-Trihydroxyflavone)	C15H10O5	30,23		269,04500	225,0550	151,0025	149,0233	117,0331	107,0126	
75[1]	Tricin (3',5'-Dimethoxy-4',5,7-trihydroxyflavone)	C17H14O7	30,41		329,06613	314,0435	313,0354	299,0199	271,0250	227,0341	
76	Chrysoeriol (3'-Methoxy-4',5,7-trihydroxyflavone)	C16H12O6	30,46		299,05556	284,0329	256,0376	227,0350	151,0024	107,0126	
77	N1,N2,N3-Triisopentenyl guanidine	C16H29N3	30,82	264,24398		196,1813	128,1187	69,0706	60,0564		[5]
78	3,3',4-Tri-O-methylellagic acid	C17H12O8	30,84		343,04540	328,0228	312,9995	297,9757	285,0046	269,9808	
79	3,3',4,4'-Tetra-O-methylellagic acid	C18H14O8	32,67	359,07670		344,0532	343,0460	329,0295	313,0346	285,0399	
80	Dihydroxy-methoxy(iso)flavone	C16H12O5	34,42	285,07630		270,0525	242,0576	153,0187	133,0652		
81	Octadecatrienol	C18H32O	45,71	265,25314		247,2422	191,1797	95,0861	83,0862	69,0706	
82	2-Hydroxystearic acid	C18H36O3	47,01		299,25863	281,2487	253,2536	251,2378	225,2219		
83	β-Sitosterol	C29H50O	49,56	415,39400		397,3836	161,1326	135,1171	95,0861	81,0705	[7]

Table S3. Chemical composition of water extract.

No.	Name	Formula	Rt	$[M + H]^+$	$[M - H]^-$	Fragment 1	Fragment 2	Fragment 3	Fragment 4	Fragment 5	Literature
1	Shikimic acid	C7H10O5	1,34		173,04500	155,0338	137,0232	111,0437	93,0330	73,0279	
2[1]	Gallic acid (3,4,5-Trihydroxybenzoic acid)	C7H6O5	2,59		169,01370	125,0230	97,0279	81,0331	79,0175	69,0330	[3]
3	Protocatechuic acid (3,4-Dihydroxybenzoic acid)	C7H6O4	5,44		153,01879	109,0280	108,0203	91,0171	81,0332		[3]
4	Galegine (Isopentenyl guanidine)	C6H13N3	6,43	128,11878		69,0706	60,0564				
5	Unidentified ellagic acid derivative	C21H10O13	11,29		469,00432	450,9955	425,0154	407,0046	379,0100	299,9914	
6	Taxifolin-O-hexoside	C21H22O12	13,85		465,10331	303,0513	285,0407	178,9977	151,0024	125,0230	
7	Putranjivain A	C46H36O31	15,67		1083,11623	935,0801	853,0730	633,0754	463,0517	300,9992	[4]
8	Brevifolincarboxylic acid or isomer	C13H8O8	17,03		291,01410	247,0245	219,0291	203,0344	191,0343	175,0390	
9	Potentillin or isomer	C41H28O26	17,26		935,07906	765,0565	463,0519	300,9991	299,9914	275,0199	
10	Procyanidin A isomer 2	C30H24O12	17,60		575,11896	539,0974	449,0885	407,0781	285,0406	125,0229	
11	Valoneic acid dilactone	C21H10O13	17,64		469,00432	450,9939	425,0153	407,0046	300,9991	299,9913	
12	Potentillin or isomer	C41H28O26	17,69		935,07906	765,0585	463,0521	300,9990	299,9913	275,0199	
13	Corilagin or isomer	C27H22O18	17,82		633,07279	463,0521	419,0623	300,9992	275,0201	169,0135	
14	Unidentified ellagic acid derivative	C21H10O13	18,49		469,00432	425,0153	300,9991	299,9913	298,9835		
15	Tellimagrandin I or isomer	C34H26O22	19,29		785,08375	633,0739	463,0515	300,9991	275,0199	169,0131	
16	Vicenin-2 (Apigenin-6,8-di-C-glucoside)	C27H30O15	19,31	595,16630		577,1554	559,1456	457,1137	325,0709	295,0606	
17[1]	Taxifolin (Dihydroquercetin)	C15H12O7	19,81		303,05048	285,0411	217,0495	175,0391	153,0179	125,0230	
18	Procyanidin A isomer 4	C30H24O12	20,17		575,11896	539,0989	449,0883	407,0797	285,0405	125,0230	
19	Ellagic acid-4-O-glucoside	C20H16O13	20,33		463,05127	300,9991	299,9913	298,9852	283,9951	257,0093	
20	Quercetin-O-hexosylhexoside	C27H30O17	20,62		625,14048	301,0352	300,0276	271,0249	255,0297	178,9971	
21	Myricetin-3'-O-glucoside	C21H20O13	21,38		479,08257	317,0303	316,0225	287,0200	271,0250	242,0213	

22	Myricetin-O-rhamnosylhexoside isomer 1	C27H30O17	21,48		625,14048	317,0300	316,0226	287,0200	271,0248	178,9977	
23	Unidentified hexahydroxydiphenoylhexose derivative	C34H26O22	21,60		785,08375	615,0649	463,0512	300,9991	275,0200	169,0131	
24[1]	Vitexin (Apigenin-8-C-glucoside)	C21H20O10	21,79	433,11347		415,1029	397,0923	379,0819	313,0710	283,0604	
25	Myricetin-O-rhamnosylhexoside isomer 2	C27H30O17	22,02		625,14048	317,0273	316,0227	287,0196	271,0241	178,9973	
26[1]	Vitexin-2''-O-rhamnoside	C27H30O14	22,13	579,17139		433,1135	415,1029	313,0710	283,0605	271,0605	
27	Taxifolin-O-pentoside	C20H20O11	22,37		435,09274	303,0512	285,0407	178,9976	151,0024	125,0230	
28	Apigenin-C-hexoside-O-pentoside	C26H28O14	22,40	565,15574		433,1133	415,1028	313,0709	283,0604	271,0603	
29	Myricitrin (Myricetin-3-O-rhamnoside)	C21H20O12	22,47		463,08765	317,0302	316,0225	287,0199	271,0249	178,9976	
30	Isovitexin (Apigenin-6-C-glucoside)	C21H20O10	22,71	433,11347		415,1025	397,0923	379,0818	313,0710	283,0604	
31	Luteolin-7-O-glucoside (Cynaroside)	C21H20O11	22,81		447,09274	327,0510	285,0407	284,0329	256,0370	133,0276	
32	Luteolin-O-rhamnosylhexoside isomer 1	C27H30O15	22,83		593,15065	447,0956	285,0407	284,0328	151,0026	133,0282	
33	Isovitexin-2''-O-rhamnoside	C27H30O14	23,01	579,17139		433,1135	415,1028	313,0710	283,0604	271,0596	
34	Hyperoside (Quercetin-3-O-galactoside)	C21H20O12	23,15		463,08765	301,0357	300,0277	271,0250	255,0298	178,9975	[6]
35	Ellagic acid-O-pentoside	C19H14O12	23,25		433,04071	300,9992	299,9913	283,9961	257,0089		
36	N1,N2-Diisopentenyl guanidine	C11H21N3	23,26	196,18138		128,1187	69,0706	60,0564			[5]
37[1]	Isoquercitrin (Quercetin-3-O-glucoside)	C21H20O12	23,39		463,08765	301,0355	300,0277	271,0249	255,0297	178,9975	
38[1]	Rutin (Quercetin-3-O-rutinoside)	C27H30O16	23,44	611,16122		465,1027	303,0503	129,0551	85,0291	71,0499	
39	Luteolin-O-rhamnosylhexoside isomer 2	C27H30O15	23,48		593,15065	447,0953	285,0407	284,0328	151,0022		
40	Eschweilenol C (Ellagic acid-4-O-rhamnoside)	C20H16O12	23,52		447,05636	300,9991	299,9913	283,9968	257,0084		
41	Ellagic acid	C14H6O8	23,75		300,99845	283,9966	257,0089	229,0136	201,0187	185,0235	[3]
42	Avicularin (Quercetin-3-O-arabinofuranoside)	C20H18O11	24,01		433,07709	301,0355	300,0277	271,0250	255,0298	178,9977	[6]
43	Mallotusinin or isomer	C41H26O25	24,22		917,06850	747,0470	615,0650	445,0415	300,9990	275,0200	
44	Apigenin-O-rhamnosylhexoside isomer 1	C27H30O14	24,38		577,15574	269,0457	268,0374	151,0030	117,0332		

45[1]	Cosmosiin (Apigenin-7-O-glucoside)	C21H20O10	24,47	433,11347		271,0604	153,0184	145,0284	119,0495		
46	Myricetin-O-galloylrhamnoside	C28H24O16	24,58		615,09861	463,0883	317,0302	316,0226	271,0249	169,0131	
47	Kaempferol-7-O-glucoside	C21H20O11	24,65		447,09274	285,0407	284,0330	255,0298	227,0345	151,0021	
48[1]	Myricetin (3,3',4',5,5',7-Hexahydroxyflavone)	C15H10O8	24,70		317,02974	271,0245	178,9976	165,0181	151,0024	137,0232	
49	Chrysoeriol-O-hexoside	C22H22O11	24,73		461,10839	446,0856	299,0575	298,0488	297,0412	283,0250	
50	Guaijaverin (Quercetin-3-O-arabinoside)	C20H18O11	24,75		433,07709	301,0353	300,0273	271,0249	255,0297	178,9976	[6]
51	Tricin-7-O-glucoside	C23H24O12	24,77		491,11896	476,0966	461,0730	328,0587	313,0357	285,0404	
52	Apigenin-O-rhamnosylhexoside isomer 2	C27H30O14	24,91		577,15574	413,0855	269,0457	268,0379	151,0025	117,0335	
53[1]	Quercitrin (Quercetin-3-O-rhamnoside)	C21H20O11	24,96		447,09274	301,0356	300,0278	271,0250	255,0298	178,9978	
54[1]	Diosmin (Diosmetin-7-O-rutinoside)	C28H32O15	24,97	609,18195		463,1241	301,0709	286,0475	129,0552	85,0291	
55	Astragalin (Kaempferol-3-O-glucoside)	C21H20O11	25,18		447,09274	285,0407	284,0328	255,0297	227,0344	151,0023	
56	Kaempferol-3-O-rutinoside (Nicotiflorin)	C27H30O15	25,34		593,15065	327,0501	285,0406	284,0329	255,0299	227,0344	
57	Unidentified ellagic acid derivative	C21H10O12	25,35		453,00940	434,9996	409,0200	391,0100	367,0093	300,9991	
58	Kaempferol-O-pentoside	C20H18O10	25,40		417,08218	285,0405	284,0328	255,0296	227,0344	151,0026	
59	3-O-Methylellagic acid	C15H8O8	26,26		315,01410	299,9912	244,0010	228,0062	200,0107		
60	Afzelin (Kaempferol-3-O-rhamnoside)	C21H20O10	26,91		431,09782	285,0406	284,0328	255,0297	227,0344	151,0025	
61[1]	Quercetin (3,3',4',5,7-Pentahydroxyflavone)	C15H10O7	27,50		301,03483	273,0409	178,9976	151,0024	121,0280	107,0124	[6]
62[1]	Naringenin (4',5,7-Trihydroxyflavanone)	C15H12O5	27,71		271,06065	227,0711	177,0179	151,0025	119,0488	107,0123	
63[1]	Luteolin (3',4',5,7-Tetrahydroxyflavone)	C15H10O6	28,37		285,03991	217,0499	199,0396	175,0390	151,0025	133,0282	
64	3,3'-Di-O-methylellagic acid	C16H10O8	28,44		329,02975	314,0073	312,9994	298,9835	270,9886		
65	Dihydroxy-methoxy(iso)flavone-O-hexoside	C22H22O10	28,59	447,12913		285,0761	270,0525	242,0570	153,0178		
66[1]	Kaempferol (3,4',5,7-Tetrahydroxyflavone)	C15H10O6	29,87		285,03991	257,0457	229,0508	213,0553	151,0026	107,0122	
67[1]	Apigenin (4',5,7-Trihydroxyflavone)	C15H10O5	30,23		269,04500	225,0550	151,0024	149,0233	117,0331	107,0125	

68[1]	Tricin (3',5'-Dimethoxy-4',5,7-trihydroxyflavone)	C17H14O7	30,41		329,06613	314,0435	313,0360	299,0199	271,0249	227,0345	
69	Chrysoeriol (3'-Methoxy-4',5,7-trihydroxyflavone)	C16H12O6	30,45		299,05556	284,0330	256,0376	227,0343	151,0035	107,0136	
70	3,3',4-Tri-O-methylellagic acid	C17H12O8	30,81		343,04540	328,0226	312,9993	297,9756	285,0042	269,9806	
71	N1,N2,N3-Triisopentenyl guanidine	C16H29N3	30,97	264,24398		196,1812	128,1186	69,0706	60,0564		[5]
72	3,3',4,4'-Tetra-O-methylellagic acid	C18H14O8	32,67	359,07670		344,0531	343,0449	329,0295	313,0348	285,0400	
73	Dihydroxy-methoxy(iso)flavone	C16H12O5	34,44	285,07630		270,0525	242,0577	153,0185	133,0653		
74	Octadecatrienol	C18H32O	45,71	265,25314		247,2423	191,1791	95,0862	83,0862	69,0706	

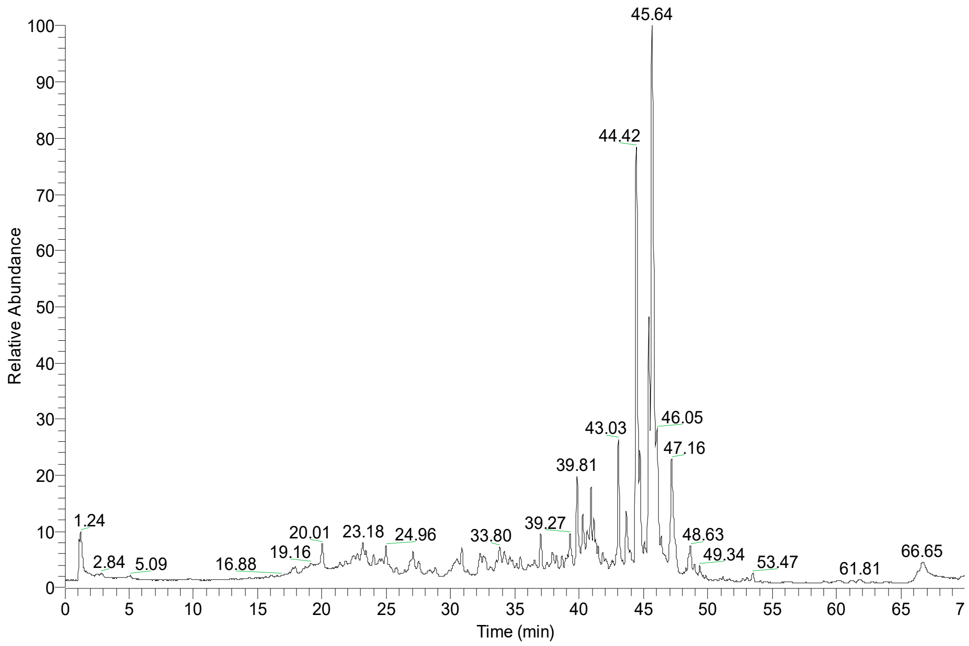

Figure S1. Total ion chromatogram of ethyl acetate extract in positive ion mode

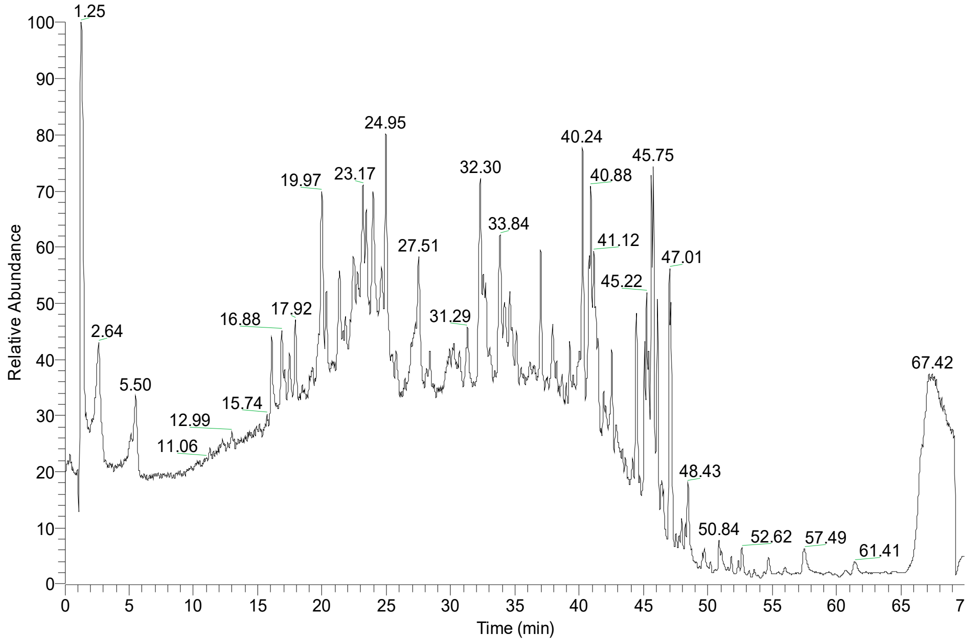

Figure S2. Total ion chromatogram of ethyl acetate extract in negative ion mode

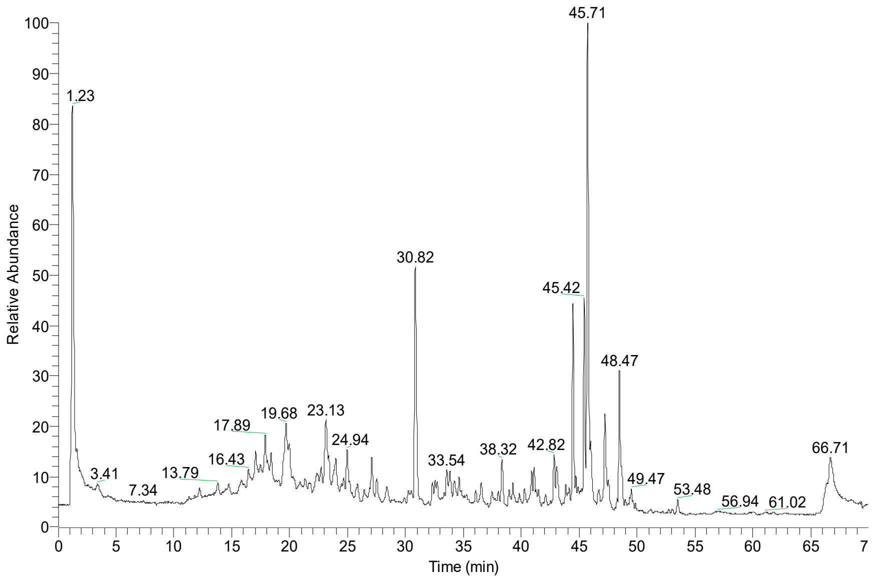

Figure S3. Total ion chromatogram of methanol extract in positive ion mode

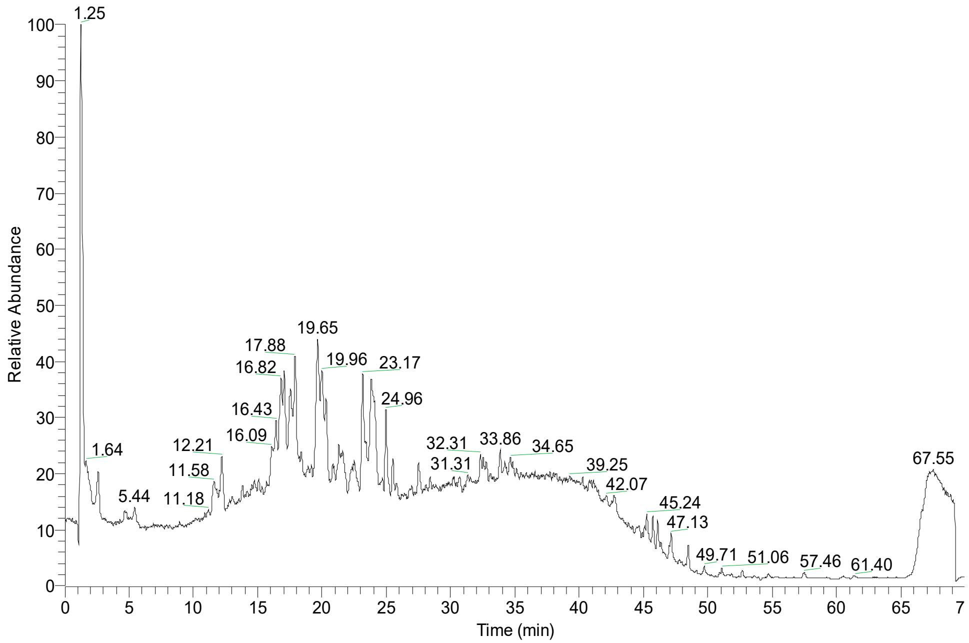

Figure S4. Total ion chromatogram of methanol extract in negative ion mode

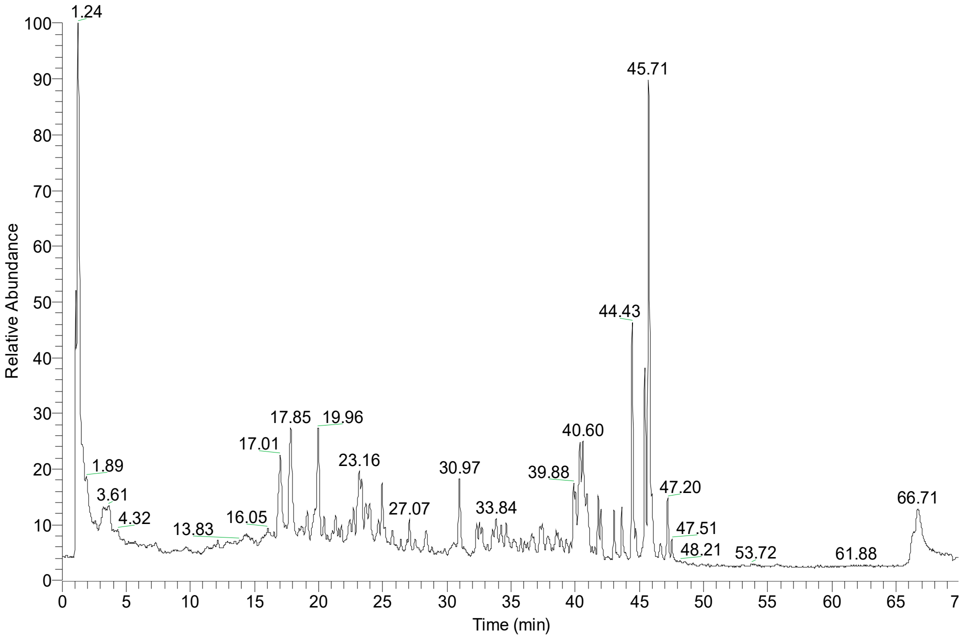

Figure S5. Total ion chromatogram of water extract in positive ion mode

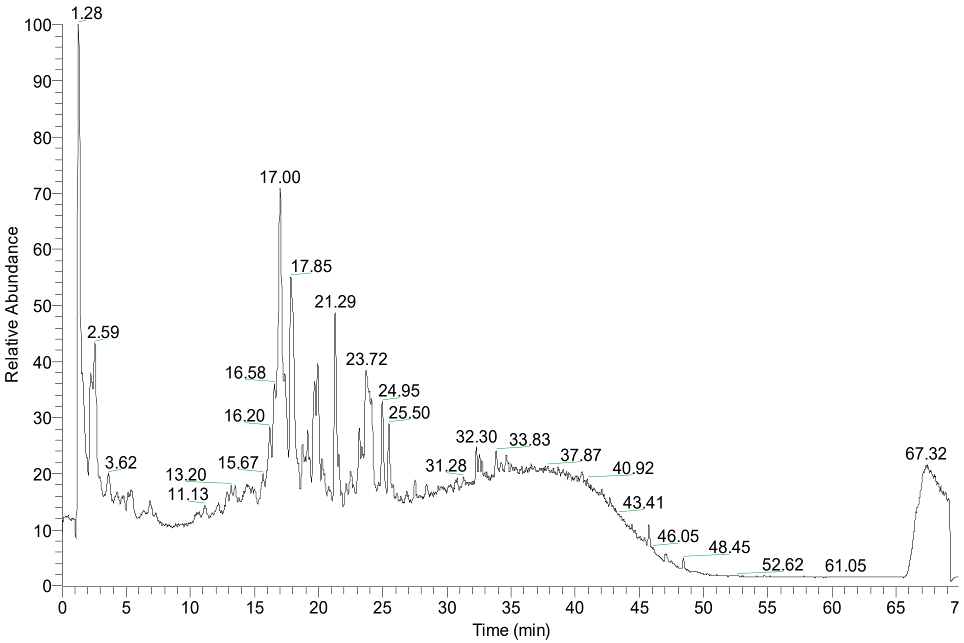

Figure S6. Total ion chromatogram of water extract in negative ion mode

1. Uysal, S.; Zengin, G.; Locatelli, M.; Bahadori, M. B.; Mocan, A.; Bellagamba, G.; De Luca, E.; Mollica, A.; Aktumsek, A., Cytotoxic and enzyme inhibitory potential of two Potentilla species (P. speciosa L. and P. reptans Willd.) and their chemical composition. *Frontiers in pharmacology* **2017,** 8, 290.
2. Grochowski, D. M.; Uysal, S.; Aktumsek, A.; Granica, S.; Zengin, G.; Ceylan, R.; Locatelli, M.; Tomczyk, M., In vitro enzyme inhibitory properties, antioxidant activities, and phytochemical profile of Potentilla thuringiaca. *Phytochemistry letters* **2017,** 20, 365-372.
3. Ogungbamila, F. O.; Samuelsson, G., Smooth muscle relaxing flavonoids from Alchornea cordifolia. *Acta Pharmaceutica Nordica* **1990,** 2, (6), 421.
4. Glensk, M.; Glinski, J. A.; Jamróz, M.; Stefanowicz, P.; Kazmierski, S., Phenolic Constituents from Alchornea castaneifolia. *Records of Natural Products* **2016,** 10, (1), 32.
5. Mavar-Manga, H.; Chapon, D.; Hoet, S.; Block, S.; De Pauw-Gillet, M.-C.; Quetin-Leclercq, J., N1, N2, N3-Trisisopentenyl guanidine and N1, N2-diisopentenyl guanidine, two cytotoxic alkaloids from Alchornea cordifolia (Schumach. & Thonn.) Müll. Arg.(Euphorbiaceae) root barks. *Natural Product Communications* **2006,** 1, (12), 1934578X0600101205.
6. Lamikanra, A.; Ogundaini, A. O.; Ogungbamila, F. O., Antibacterial constituents of Alchornea cordifolia leaves. *Phytotherapy Research* **1990,** 4, (5), 198-200.
7. Mavar-Manga, H.; Haddad, M.; Pieters, L.; Baccelli, C.; Penge, A.; Quetin-Leclercq, J., Anti-inflammatory compounds from leaves and root bark of Alchornea cordifolia (Schumach. & Thonn.) Müll. Arg. *Journal of Ethnopharmacology* **2008,** 115, (1), 25-29.
